# Fungal-Induced Cell Cycle Impairment, Chromosome Instability and Apoptosis via Differential Activation of NF-κB

**DOI:** 10.1371/journal.ppat.1002555

**Published:** 2012-03-01

**Authors:** Mariem Ben-Abdallah, Aude Sturny-Leclère, Patrick Avé, Anne Louise, Frédérique Moyrand, Falk Weih, Guilhem Janbon, Sylvie Mémet

**Affiliations:** 1 Institut Pasteur, Unité de Mycologie Moléculaire, Département Infection et Epidémiologie, Paris, France; 2 CNRS, URA3012, Paris, France; 3 Institut Pasteur, Unité d'Histopathologie, Département Infection et Epidémiologie, Paris, France; 4 Institut Pasteur, Plateforme d'Imagerie Dynamique, Paris, France; 5 Institut Pasteur, Unité des Aspergillus, Département de Parasitologie et Mycologie, Paris, France; 6 Leibniz-Institute for Age Research - Fritz-Lipmann-Institute, Research Group Immunology, Jena, Germany; University of Massachusetts Medical School, United States of America

## Abstract

Microbial pathogens have developed efficient strategies to compromise host immune responses. *Cryptococcus neoformans* is a facultative intracellular pathogen, recognised as the most common cause of systemic fungal infections leading to severe meningoencephalitis, mainly in immunocompromised patients. This yeast is characterized by a polysaccharide capsule, which inhibits its phagocytosis. Whereas phagocytosis escape and macrophage intracellular survival have been intensively studied, extracellular survival of this yeast and restraint of host innate immune response are still poorly understood. In this study, we have investigated whether *C. neoformans* affected macrophage cell viability and whether NF-κB (nuclear factor-κB), a key regulator of cell growth, apoptosis and inflammation, was involved. Using wild-type (WT) as well as mutant strains of *C. neoformans* for the pathogen side, and WT and mutant cell lines with altered NF-κB activity or signalling as well as primary macrophages for the host side, we show that *C. neoformans* manipulated NF-κB-mediated signalling in a unique way to regulate macrophage cell fate and viability. On the one hand, serotype A strains reduced macrophage proliferation in a capsule-independent fashion. This growth decrease, which required a critical dosage of NF-κB activity, was caused by cell cycle disruption and aneuploidy, relying on fungal-induced modification of expression of several cell cycle checkpoint regulators in S and G2/M phases. On the other hand, *C. neoformans* infection induced macrophage apoptosis in a capsule-dependent manner with a differential requirement of the classical and alternative NF-κB signalling pathways, the latter one being essential. Together, these findings shed new light on fungal strategies to subvert host response through uncoupling of NF-κB activity in pathogen-controlled apoptosis and impairment of cell cycle progression. They also provide the first demonstration of induction of aneuploidy by a fungal pathogen, which may have wider implications for human health as aneuploidy is proposed to promote tumourigenesis.

## Author Summary

*Cryptococcus neoformans*, the only encapsulated pathogenic yeast, is responsible for severe opportunistic meningoencephalitis mostly in immunocompromised patients. It is a facultative intracellular pathogen and, as such, has the ability to survive intra- and extracellularly. Whereas interactions of *C. neoformans* with macrophages, especially its phagocytosis escape and intracellular survival, have been intensively studied, little is known about other schemes allowing extracellular survival of this yeast and restraint of host innate immune response. Here, we report that *Cryptococcus neoformans* compromised macrophage viability in two ways. Firstly, fungal infection elicited a strong decrease in macrophage proliferation in a capsule-independent fashion. This inhibition was subsequent to fungal-induced cell cycle disruption and chromosome aberrations (aneuploidy), a phenomenon commonly triggered by bacteria or viruses but for the first time described for a fungus. Secondly, this pathogen promoted apoptosis in a capsule-dependent manner. Our findings unravel a new process by which a fungal pathogen dampens the immune response using uncoupled activity of NF-κB (a key regulator of cell growth, apoptosis and inflammation) in fungal-induced apoptosis and inhibition of cell proliferation. This may have larger implications for human health, as animal models suggest that aneuploidy promotes tumourigenesis.

## Introduction

*Cryptococcus neoformans* is a facultative intracellular pathogen that is the most common cause of systemic fungal infections leading to meningoencephalitis in immunocompromised patients, and notably in people infected with HIV [1], [2]. This saprophytic basidiomycete fungus is characterized by the presence of a polysaccharide capsule, composed of glucuronoxylomannan (GXM), galactoxylomannan (GalXM) and mannoproteins. The capsule constitutes the main virulence factor of *C. neoformans* and inhibits its phagocytosis [3]–[5]. Infection by *C. neoformans* is thought to result from its inhalation as basidiospores and usually leads to asymptomatic pneumonia, followed by a latent phase that can last many years [6]. When immunodepression arises, reactivated yeasts disseminate into the bloodstream, reach the central nervous system and cause fatal meningoencephalitis if left untreated. In the pathogenesis of cryptococcosis, macrophages play a major defence role [7]–[11].

To evade the host immune system and macrophage-mediated killing in particular, *C. neoformans* has developed several stratagems. Among those, its phagocytosis by innate immune cells is inhibited through both capsule-dependent (for review see [12]) and capsule-independent mechanisms [13], [14]. Once phagocytosed, *C. neoformans* has the ability to exit the macrophage through a mechanism that does not kill the host cell thereby avoiding inflammation [15]. In addition, *C. neoformans* can survive inside the phagolysosome and macrophage serves as a site for both fungal replication and reservoir during latency [16], [17]. Remarkably, macrophages do not spontaneously phagocytose *C. neoformans*, as they do for other microbes and human pathogenic fungi. They require its opsonisation, a process involving complement or antibodies, production of which takes several days in the mouse [18]. Without opsonisation, and therefore during the early phase of infection prior to antibody generation and in tissues with low levels of complement, very little production of inflammatory cytokines is triggered by *C. neoformans* [19], [20]. Programmed cell death (PCD) of immune cells may constitute another way to limit inflammation, as apoptotic inflammatory cells fail to release their proinflammatory and histotoxic substances. Indeed, purified capsule components, GXM and GalXM polysaccharides, have been shown to induce apoptosis of T cells [21], [22] as well as macrophages [23], [24]. However, the precise mechanisms involved in macrophage PCD are not fully elucidated and little is known about other strategies elicited by unopsonised *C. neoformans* to promote immune evasion, especially about a direct effect of this pathogen on viability and growth of the macrophage itself.

NF-κB is a ubiquitous transcription factor with post-translationally regulated activity, which plays a pivotal role in inflammation, immunity, cell growth and apoptosis through the control of expression of major immunomodulatory, cell proliferation and death regulatory genes [25]–[27]. Five mammalian NF-κB subunits that can form homo- or hetero-dimeric combinations have been identified: p50, p52, p65/RelA, c-Rel and RelB. In most cells, NF-κB dimers are sequestered in the cytoplasm by interaction with inhibitory proteins, the IκBs, namely IκBα, IκBβ, IκBε, p105 and p100. The nuclear translocation of NF-κB is regulated by two prevailing activation pathways [28]. The classical/canonical one, induced by engagement of cytokines as well as death and pattern recognition receptors, depends on the IKK complex, which is composed of two catalytic subunits IKKα/1 and IKKβ/2 and a regulatory subunit NEMO/IKKγ. Upon stimulation, the IKK complex triggers phosphorylation of two Ser residues within the N-terminus of the IκBs, leading to their ubiquitination and degradation by the proteasome thereby releasing NF-κB dimers to the nucleus. In macrophages, activation of classical NF-κB dimers is predominantly controlled by IKK2 [29], [30]. The alternative pathway, induced by certain members of the TNF family such as lymphotoxin-β or CD40L [31], [32], is NEMO- and IKK2- independent, and specifically involves phosphorylation of IKK1 by NF-κB-Inducing Kinase (NIK). This in turn triggers phosphorylation of p100, the main RelB inhibitor, and its processing to p52 thereby freeing RelB/p52 heterodimers. These two pathways are complexly interconnected and activation of the alternative pathway may be regulated by the induction of p50/p65, or Rel complexes [33].

In this study, we have explored whether *C. neoformans* like other microbes, such as bacteria or viruses, uses inhibition of cell viability to restrain host immune response and asked how NF-κB might control fungal-regulation of macrophage survival. We report that unopsonised *C. neoformans* elicited apoptosis of macrophages as well as repressed cell proliferation through disruption of macrophage cell cycle via the modification of expression of an array of cell cycle checkpoint regulators. We further show that the classical and alternative pathways of NF-κB activation were differently required for these processes. Collectively, these findings shed light on a new fungal strategy to evade host response through uncoupling of NF-κB activity in pathogen-induced apoptosis and cell cycle progression impairment. They also disclose for the first time fungal-induced aneuploidy.

## Results

### Fungal infection decreased cell viability in a capsule-independent manner via reduced cell proliferation

**Figure 1:**
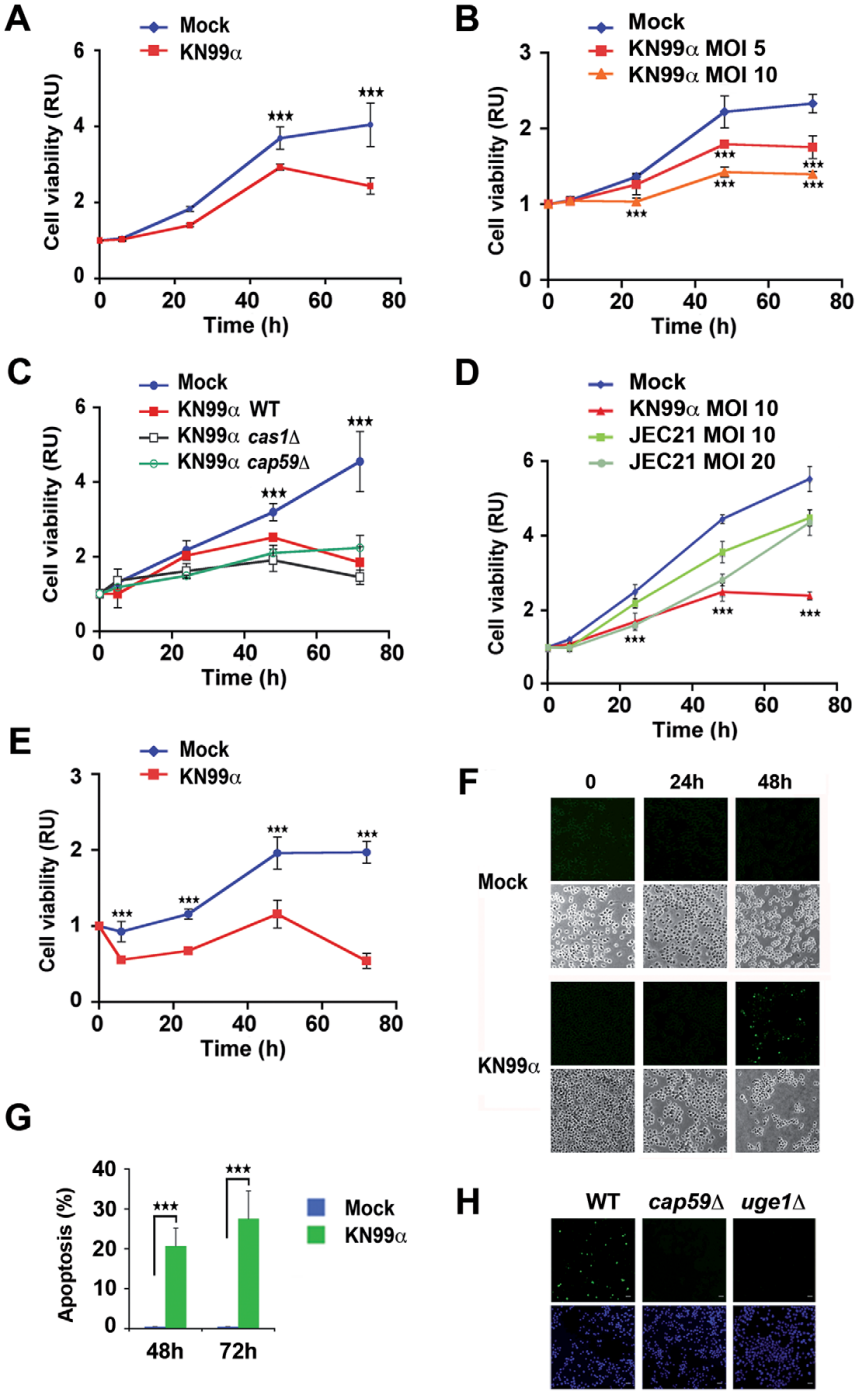
Fungal infection decreases macrophage cell viability in a capsule-independent fashion and promotes capsule-dependent cell apoptosis. (**A**) Time-course of cell viability of mock-treated or *C. neoformans*-infected (KN99α) J774 cells was assessed using a commercial viability assay generating a luminescent signal directly proportional to the amount of ATP present in metabolically active cells. Results are presented as fold relative to the cell viability in mock-treated cells at time 0. Data are mean ± s.e.m. (n = 6). ***, *P*<0.001. (**B**) Time-course of cell viability of J774 cells mock-treated or infected at different M.O.I. with *C. neoformans* (KN99α) assessed as mentioned above. Data are mean ± s.e.m. (n = 6). ***, *P*<0.001, compared with the mock-treated cells. (**C**) Time-course of cell viability of J774 cells mock-treated or infected by WT (KN99α) or capsule mutant *C. neoformans* strains (*cas1*D, *cap59*D), evaluated as mentioned above. Data are mean ± s.e.m. (n = 6). ***, *P*<0.001, compared with mock-treated cells. (**D**) Time-course of cell viability of J774 cells mock-treated or infected at different M.O.I. with serotype D *C. neoformans* (JEC21) assessed as mentioned above. Data are mean ± s.e.m. (n = 6). ***, *P*<0.001, compared with the mock-treated cells or with the JEC21-infected cells at M.O.I. 10 or with the JEC21-infected cells at M.O.I. 20, last time-point only. (**E**) Time-course of cell viability of BMDM cells mock-treated or *C. neoformans*-infected assessed as mentioned above. BMDM were cultured in presence of 30% CSF-conditioned medium from L929 cells and used directly without synchronization by M-CSF starvation. Data are mean ± s.e.m. (n = 6). ***, *P*<0.001, compared with mock-treated cells. (**F**) Representative TUNEL staining of J774 mock-treated or treated with *C. neoformans* (KN99α) at the indicated times (upper panels) and phase contrast microscopy images of the same cells (lower panels). (**G**) Quantification of the number of TUNEL^+^ BMDM mock-treated or *C. neoformans*-infected (KN99α) at the indicated time-points. BMDM were cultured in presence of 30% CSF-conditioned medium from L929 cells and used directly without synchronization by M-CSF starvation. Values are expressed as % of TUNEL^+^ cells for the total number of cells. Data are mean ± s.e.m. (n = 2). ***, *P*<0.001. (**H**) Representative TUNEL (top panels) and DAPI (bottom panels) staining of 48 h-infected J774 cells with wild-type (KN99α) or mutant *C. neoformans* strains (*cap59*D, lacking GXM the major capsule component and devoid of polysaccharide capsule or *uge1*D, lacking GalXM). Scale bar 20 µm.

**Figure 2:**
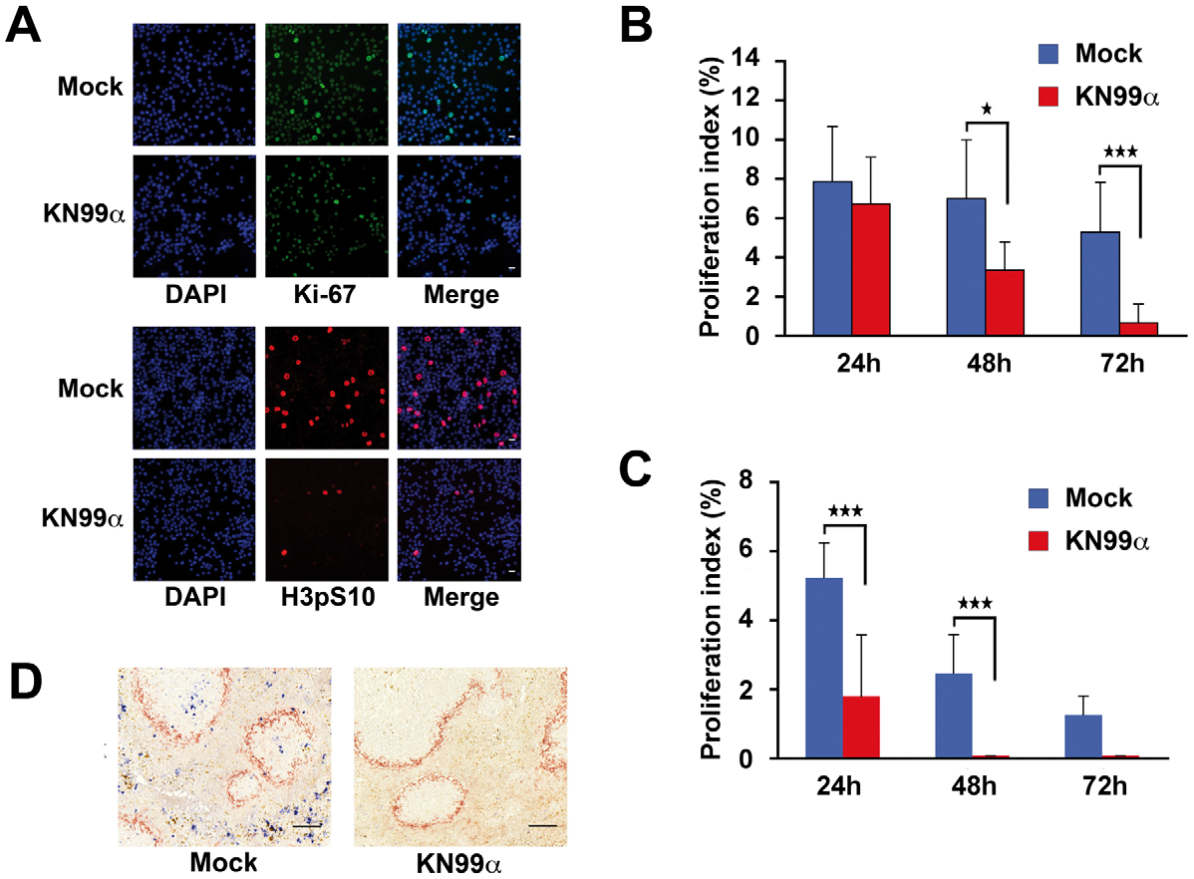
Fungal infection decreases cell proliferation. (**A**) Ki-67 or Phospho-H3 (H3pS10) immunofluorescence and DAPI staining of mock-treated or *C. neoformans*-infected (KN99α) J774 cells 48 h p.i.. Scale bar 20 µm. (**B**) Quantification of the number of Phospho-H3^+^ nuclei of mock-treated or *C. neoformans*-infected (KN99α) J774 cells at the indicated time-points. Proliferation index (%) is the number of Phospho-H3^+^ nuclei for the total number of cell nuclei (blue). Data are mean ± s.e.m. (n = 3). *, *P*<0.05. ***, *P*<0.001. (**C**) Quantification of the number of Phospho-H3^+^ nuclei of mock-treated or *C. neoformans*-infected (KN99α) BMDM at the indicated time-points. BMDM were cultured in presence of 30% CSF-conditioned medium from L929 cells and used directly without synchronization by M-CSF starvation. Proliferation index (%) is the number of Phospho-H3^+^ nuclei for the total number of cell nuclei. Data are mean ± s.e.m. (n = 3). ***, *P*<0.001. (**D**) Immunohistocytochemical analysis with antibodies against Phospho-H3 (H3pS10) (Blue) and the macrophage marginal zone marker MOMA-1 (red) from spleen sections of mock-treated or KN99α *C. neoformans*-infected κB-*lacZ* mice 3 d p.i.. Scale bar 100 µm. Representative panels are shown from n = 6 mice.

To explore the effects of unopsonised *C. neoformans* on macrophage viability, we first verified that in our conditions of infection, phagocytosis of *C. neoformans* by J774 macrophage-like cells was negligible. Two serotype A strains were studied in parallel, the wild-type (WT) (KN99α) and an acapsular mutant (*cap59*D, devoid of GXM the major capsule component [34]), expected to be more phagocytosed [35]. Indeed, a maximum of 4% of phagocytosis was observed for the WT strain with 1–3 yeasts ingested per cell after 48 h of infection. For the acapsular strain, phagocytosis although higher, did not exceed 15.7%, with in that case 1–20 yeasts internalized per cell (Figure S1). This indicated that in our experimental settings, unopsonised WT and even acapsular *C. neoformans* remained mostly extracellular. We next examined the effect of unopsonised *C. neoformans* infection on macrophage cell viability. We found a significant decrease in viability of J774 macrophage-like cells, measured by ATPmetry with unopsonised KN99α from 48 h post-infection (p.i.) onward (Figure 1A). This decline of viability was dose-dependent (Figure 1B) but independent of the capsule as assessed by a similar effect of WT (KN99α) and capsule mutant strains of *C. neoformans* (*cap59*D, lacking GXM; *cas1*D, missing O-acetylation of the capsule [36]; suppressors of *uge1*D, devoid of GalXM with a doubling time similar to that of WT at 37°C [36]) (Figure 1C and Figure S2). However, a serotype D strain of *C. neoformans* led to a mild effect even at high M.O.I. (20) with an overall profile of viability parallel to that of mock-treated cells, and therefore completely different from that of serotype A-infected cells (Figure 1D). This fungal-induced inhibition of viability required both viable yeasts and pathogen-cell contact as revealed by the analogous behaviour of heat-inactivated *C. neoformans* and mock-treated J774 cells, as well as the absence of effect of J774 cell infection by KN99α in transwells (Figure S3). Remarkably, fungal-induced reduction of viability also occurred in primary macrophagic cells such as bone marrow derived macrophages (BMDM) (Figure 1E). Since cell viability reflects the balance between cell proliferation and death, we then investigated whether apoptosis was elicited by *C. neoformans* in macrophages. TUNEL^+^ cells were detected 48 h p.i. with the WT strain of *C. neoformans* in 11.6% of J774 macrophage-like cells and in 20.7% of BALB/c BMDM and persisted 72 h p.i. (Figure 1F, G). Capsule mutant strains of *C. neoformans* (*cap59*D lacking GXM, or *uge1*D devoid of GalXM) did not trigger any apoptosis, indicating an essential function of these capsular components (Figure 1H). Then, we assessed whether macrophage cell growth was inhibited by fungal infection. Indeed, significantly reduced numbers of mitotic J774 macrophage-like cells were detected by immunocytochemical analysis using two classical proliferative markers Ki-67 and phospho-histone H3 (Ser10) from 48 h p.i. onward (Figure 2A, B). Such fungal-induced diminution of proliferation also operated in BMDM (Figure 2C) and more generally in the spleen *in vivo* 3 d p.i. of mice challenged with *C. neoformans* in a model that mimics the systemic infection in humans [37], as revealed by phospho-histone H3 immunohistochemistry (Figure 2D).

### Fungal-induced cell growth inhibition reflected cell cycle impairment and aneuploidy

**Figure 3:**
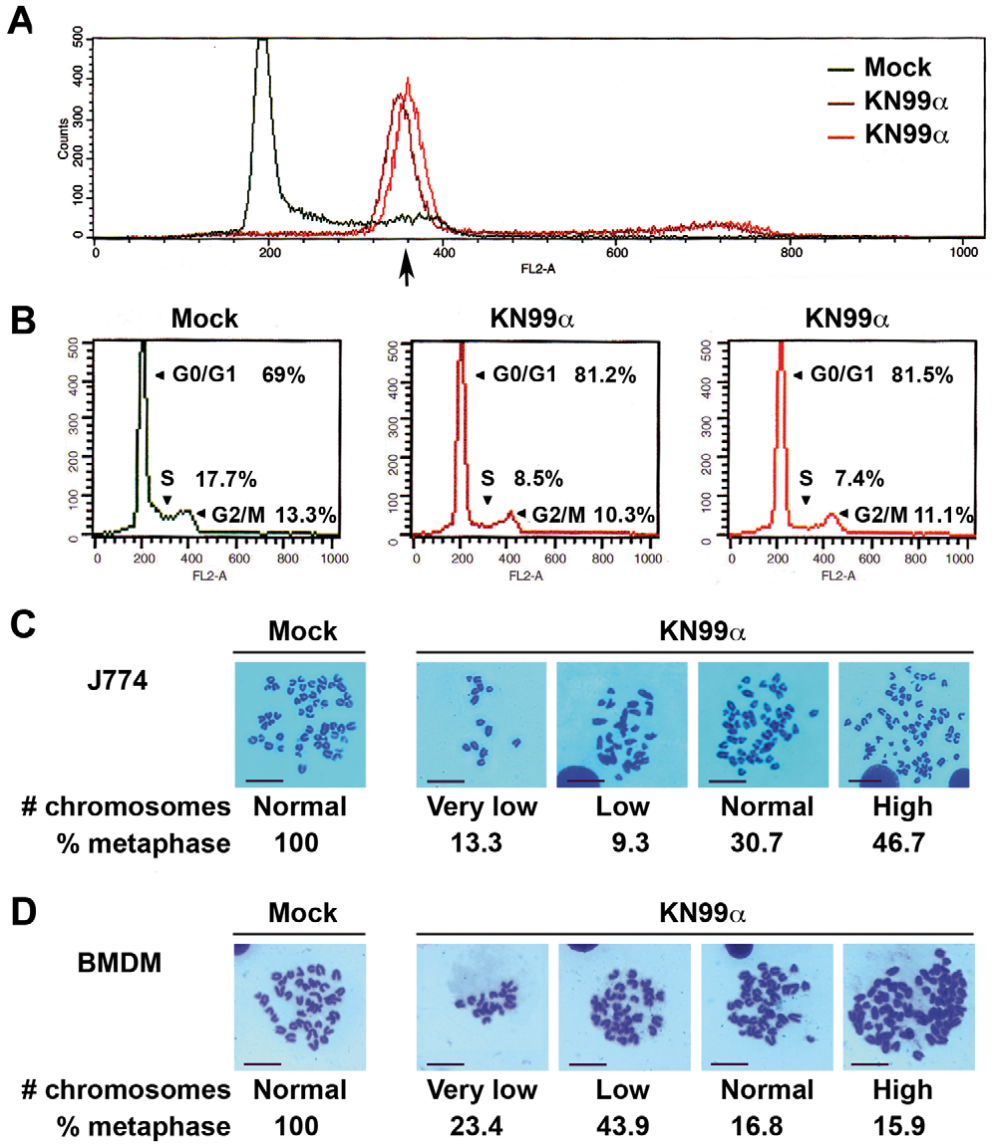
Fungal-induced cell growth inhibition reflects cell cycle impairment and aneuploidy. (**A**) Representative flow cytometry histograms of cell cycle distribution of mock-treated or *C. neoformans*-infected (KN99α, 2 independent infections) J774 cells after 48 h, assessed by propidium iodide incorporation (n = 6). X-axis shows intensity of fluorescence and Y-axis number of cells. 30000 total events were acquired per sample with identical parameters of acquisition for all samples and data were analysed as described in Protocol S1. Arrow indicates an increase in DNA content. (**B**) Quantification of cells shown in (**A**) in G0/G1, S and G2/M phases by flow cytometry and analysis with the CellQuest software, with peak of G0/G1 arbitrarily positioned upon acquisition at 200 on the linear scale of FL2-A X-axis for each sample (30000 total events acquired). (**C**) Representative images of Giemsa-stained metaphase chromosomes from mock-treated or 48 h-infected J774 (n = 75 for infected and 23 for mock-treated cells). (**D**) Representative images of Giemsa-stained metaphase chromosomes from mock-treated or 48 h-infected BMDM (n = 107 for infected and 18 for mock-treated cells). BMDM were cultured in presence of 30% CSF-conditioned medium from L929 cells and used directly without synchronization by M-CSF starvation. Values indicated are % of metaphases with the following number of chromosomes: Very low (8–27), low (29–36) and high (50–114). Scale bar 50 µm.

**Figure 4:**
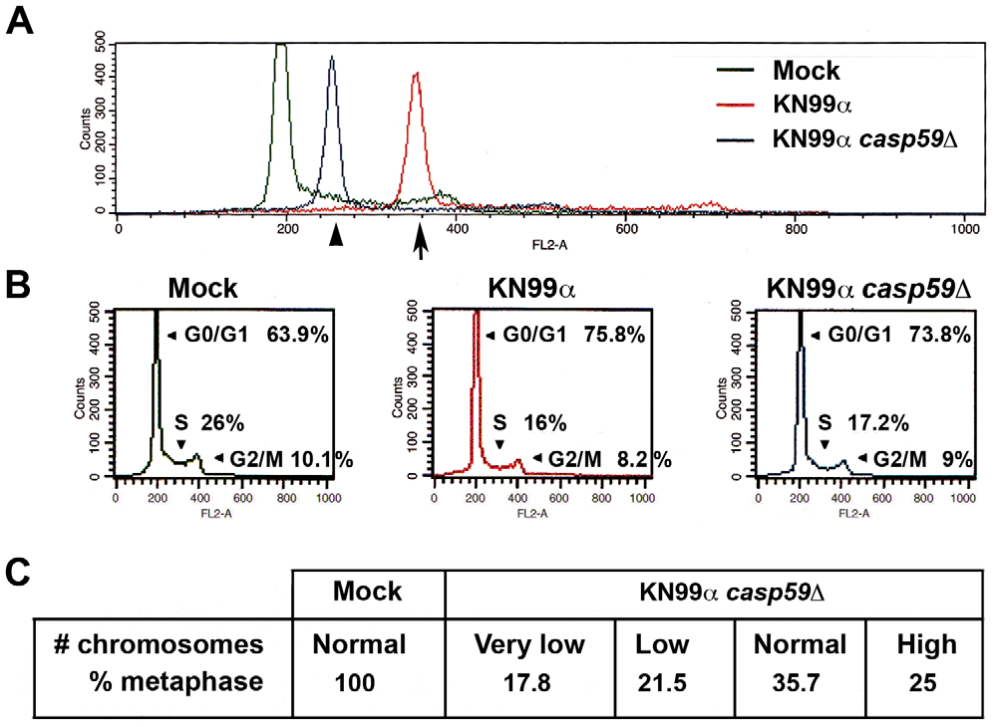
Cell cycle impairment and aneuploidy are also triggered by acapsular strain. (**A**) Representative flow cytometry histograms of cell cycle distribution of J774 cells, mock-treated or infected for 48 h with WT (KN99α) or acapsular mutant (*cap59*D) *C. neoformans* strains, assessed by propidium iodide incorporation (n = 3). X-axis shows intensity of fluorescence and Y-axis number of cells. 30000 total events were acquired per sample with identical parameters of acquisition for all samples and data were analysed as described in Protocol S1. Arrow indicates an increase in DNA content. (**B**) Quantification of cells shown in (**A**) in G0/G1, S and G2/M phases by flow cytometry and analysis with the CellQuest software, with peak of G0/G1 arbitrarily positioned upon acquisition at 200 on the linear scale of FL2-A X-axis for each sample (30000 total events acquired). (**C**) Table established from chromosome spreads of mock-treated J774 cells or of cells infected for 48 h with acapsular mutant strain (*cap59*D). n = 29 for infected and 18 for mock-treated cells. Values indicated are % of metaphases with the following number of chromosomes: Very low (8–27), low (29–36) and high (50–114).

We then asked whether this decrease of proliferation might originate from a disruption of the host cell cycle by *C. neoformans*. Representative graphs of cell cycle distribution determined by propidium iodide staining and flow cytometry showed a clear shift of the peak corresponding to J774 cells (Figure 3A, Figure S4A) or BMDM (Figure S4B) in G0/G1 48 or 72 h p.i.. This shift, ascertained by invariance of a chick erythrocyte control standard peak (Figure S5), indicated an augmentation of DNA content, which varied in the range of 1.5 to 2 diploid DNA content. In addition, quantification of cells in the various phases of the cell cycle disclosed a reduction in proportion of cells both in S and G2/M upon infection (Figure 3B), in accordance with the immunocytochemistry data shown above (Figure 2A, B). Altogether, these results revealed that *C. neoformans* infection impaired macrophage cell cycle together with a modification of its DNA content. To further confirm an effect of this fungal pathogen on cell ploidy, metaphase preparations from untreated (mock) or 48 h-infected J774 cells or BMDM were analyzed (Figure 3C, D). Overall, we noticed a strong decrease (about 3- to 10-fold less) in the total number of metaphases for both J774 or BMDM infected cells compared to mock-treated ones, consistently with the cell cycle defects unveiled above. When focusing on metaphasic chromosomes, we observed that infection by *C. neoformans* induced a variation in chromosome number in both J774 cells and BMDM. We next asked whether these effects on cell cycle and ploidy were also triggered by an acapsular strain of *C. neoformans*. Representative graphs of cell cycle distribution determined by propidium iodide staining and flow cytometry disclosed, as for WT *C. neoformans*, a clear shift of the peak corresponding to J774 cells in G0/G1 infected for 48 h by the acapsular strain (*cap59*D) (Figure 4A). In addition, quantification of cells in the various phases of the cell cycle revealed a similar decrease of the number of infected cells in S and G2/M phases by both strains (Figure 4B). Consistently, metaphase preparations showed a modification of chromosome numbers in J774 cells infected by the acapsular strain (*cap59*D) (Figure 4C). Thus in macrophages, numerical changes in whole chromosomes (aneuploidy) were induced by a fungal pathogen.

### Infection by *C. neoformans* induced NF-κB activation both *in vitro* and *in vivo*

**Figure 5:**
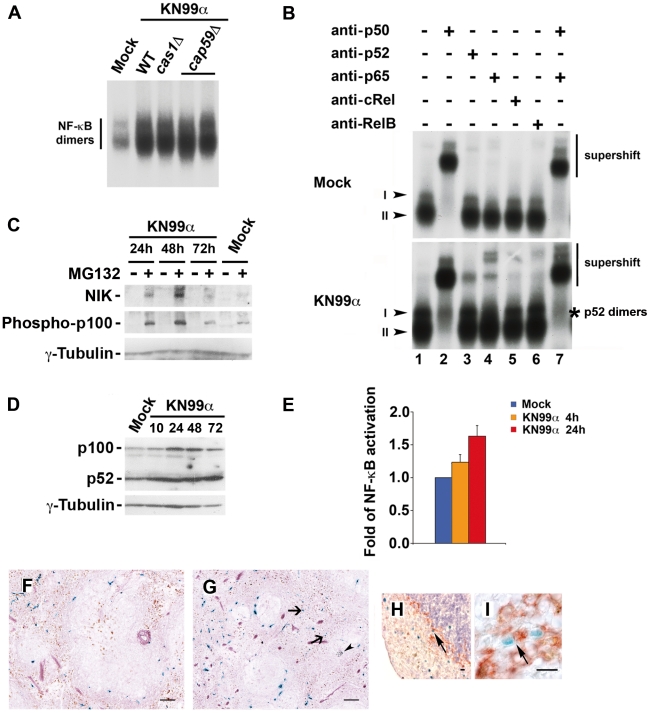
Infection by *C. neoformans* induces NF-κB activation both *in vitro* and *in vivo*. (**A**) Five µg of nuclear extracts from J774 cells mock-treated or infected for 24 h by WT (KN99α) or mutant *C. neoformans* strains (*cas1*D, *cap59*D) were analyzed by EMSA for their ability to bind to a double-stranded oligonucleotide corresponding to a canonical κB site from the MHC class I gene (n = 2). (**B**) Nuclear extracts (5 µg) from J774 cells mock-treated or infected for 24 h by WT *C. neoformans* strain (KN99α) were preincubated with preimmune serum or sera directed against each member of the NF-κB family alone or in combination, and analyzed by EMSA. Arrowheads point out the main complexes (I, II) visualized. * indicates specific p52-containing dimers (alternative pathway) (n = 3). (**C**) J774 cells were mock-treated or infected for the indicated times by WT *C. neoformans* strain (KN99α); 3 h before recovery and preparation of whole cell extracts, cells were pretreated (+) or not (−) with MG132 (20 µM); 100 µg of total protein extracts were then subjected to immunoblotting with anti-NIK or anti-Phospho-p100 or anti-γ-tubulin (internal loading control) antibodies (n = 2). (**D**) 25 µg of total protein extracts from J774 cells mock-treated or infected for the indicated times (h) by WT *C. neoformans* strain (KN99α) were analyzed by western blot with antibodies against p100 or γ-tubulin as an internal loading control. (**E**) J774 cells were transfected with a NF-κB-luciferase reporter plasmid containing the site from the enhancer of the Ig κ light chain gene (Igκ) together with an EF1-*lacZ* normalization vector. After 24 h, cells were mock-treated or infected with *C. neoformans* (KN99α) for the indicated times. Results are presented as fold relative to the activity in mock-treated cells. Data are mean ± s.e.m. (n = 3). (**F**) X-gal and Gomori-Grocott staining of a spleen section from control κB-*lacZ* mice counterstained with safranin. (**G**) X-gal and Gomori-Grocott staining of a spleen section from KN99α *C. neoformans*-infected κB-*lacZ* mice counterstained with safranin 3 d p.i.. Arrows, β-galactosidase^+^ cells (blue); arrowhead, yeasts (black). (**H**) X-gal staining and immunohistocytochemical analysis with antibody against the marginal zone macrophage marker MOMA-1 (red) from a spleen section of *C. neoformans*-infected κB-*lacZ* mice counterstained with hematoxylin 3 days p.i.. Arrow, double-stained β-galactosidase^+^MOMA-1^+^ cell. (**I**) X-gal staining and immunohistocytochemical analysis with antibody against the pan macrophage marker F4/80 (red) from a spleen section of *C. neoformans*-infected κB-*lacZ* mice 3 d p.i.. Arrow, double-stained β-galactosidase^+^F4/80^+^ cell. Scale bar 100 µm (**F**, **G**). Scale bar 10 µm (**H**, **I**). Representative panels are shown from n = 6 mice.

Given the seminal role of NF-κB in growth control and survival, we next investigated whether *C. neoformans* induced NF-κB activity. EMSA analysis disclosed that both WT (KN99α) and capsule mutant strains of *C. neoformans* led to a strong increase in NF-κB binding activity, represented by two major complexes, I and II, in J774 murine macrophage-like cells (Figure 5A, B). Supershift experiments identified complex II as p50 homodimers. Pathogen-induced complex I consisted of p50-containing heterodimers including p50/p65 as well as specific p52-containing dimers (Figure 5B). NIK is a serine-threonine kinase that is critical for the induction of the IKK1-dependent processing of p100. Western blot analysis of total J774 protein extracts revealed that NIK expression levels were induced by WT *C. neoformans* (KN99a), especially at 24 and 48 h p.i., but only when proteasome was inhibited by MG132 treatment (Figure 5C). This indicated that the steady-state levels of NIK were elevated upon infection of J774 cells, and suggested that, although NIK might be abundantly produced in J774 cells by *C. neoformans* infection, it was rapidly degraded by the proteasome. The levels of NIK expression correlated well with those of phosphorylated p100 (Figure 5C). *C. neoformans* also enhanced p100 levels after 24 and 48 h of infection and, in accordance with the EMSA and NIK/Phospho p100 data, induced processing of the p100 to p52, resulting in increased p52 levels from 24 h p.i. onward (Figure 5D). Thus, both the alternative and the classical pathways of NF-κB activation were induced by *C. neoformans* in macrophages. We then asked whether this induction of NF-κB DNA binding activity was associated with an increase in NF-κB-dependent gene expression. In a NF-κB-reporter assay, *C. neoformans* infection of transfected J774 cells elicited after 4 h a small rise in NF-κB activation, which was amplified after 24 h (Figure 5E). When challenging *in vivo* κB-*lacZ* reporter transgenic mice by *C. neoformans* in a model that mimicked systemic infection in humans, increase in β-galactosidase^+^ cells, including macrophages, was observed 3 d p.i. in the spleen (Figure 5F–I) and correlated with high fungal burden (2.7×10^3^ CFU±1.0). Taken together these results demonstrated *C. neoformans*-induced NF-κB-transactivation both *in vitro* and *in vivo*.

### Both classical and alternative NF-κB pathways contributed to fungal-induced cell apoptosis, the alternative one being essential

**Figure 6:**
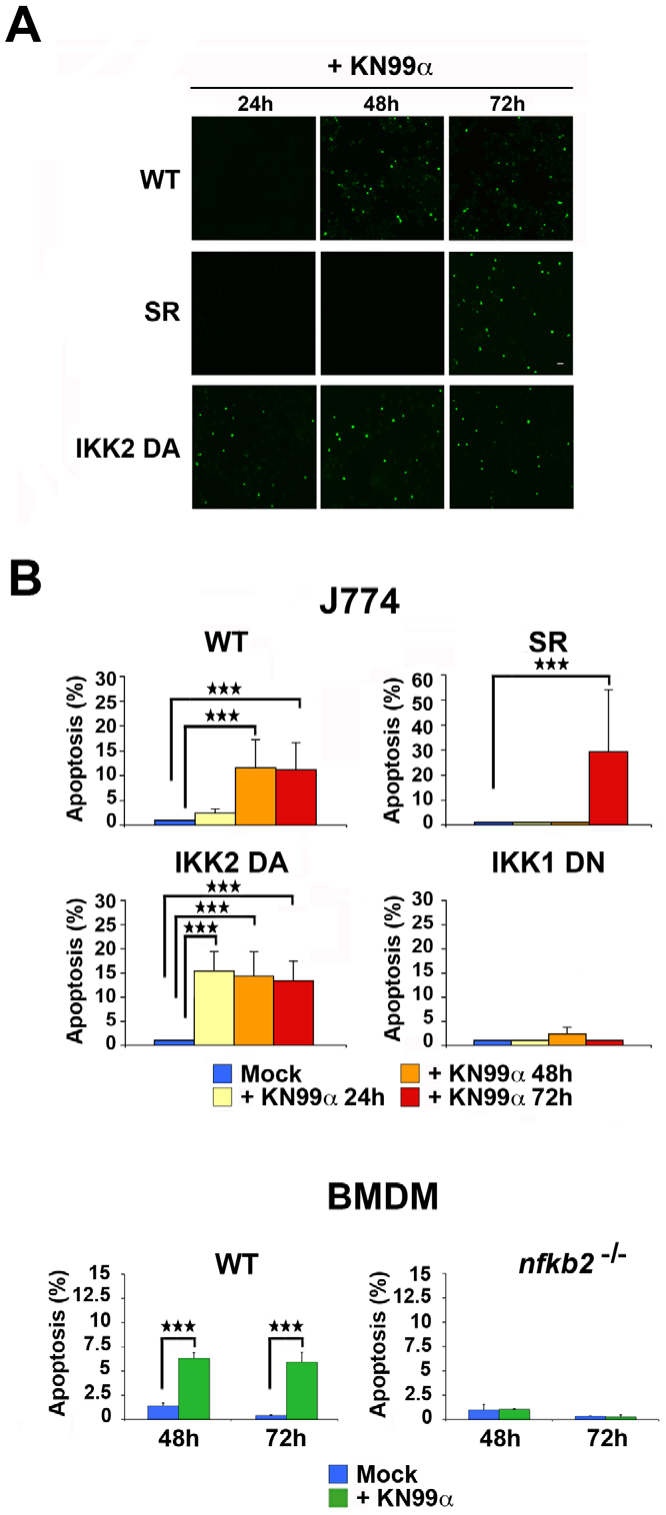
Both the classical and the alternative pathways of NF-κB activation contribute to fungal-induced apoptosis of macrophages, the alternative one being essential. (**A**) TUNEL staining of SR or IKK2 DA mutant stable clones versus WT J774 cells infected with *C. neoformans* (KN99α) for 24, 48 or 72 h. Scale bar 20 µm. (**B**) Histogram showing the quantification of apoptosis by TUNEL assay for WT J774 and stable NF-κB-modulated clones mock-treated or infected by *C. neoformans* (KN99α) at different time-points and for C57BL/6 control or *nfκb2* ^−/−^ BMDM at 48 and 72 h p.i.. Results are expressed as mean ± s.e.m of % of TUNEL^+^ cells relative to total cell number (n = 3). ***, *P*<0.001.

To decipher the role of NF-κB in these processes, stable J774 clones with constitutively altered NF-κB activity or IKK1- or IKK2-dependent signalling (Figure S6) were generated by overexpression of either the super-repressor/IκBα-AA (SR) or a kinase-dead mutant of IKK2 (IKK2 DN) or a kinase-active mutant of IKK2 (IKK2 DA) or a kinase-dead mutant of IKK1 (IKK1 DN). To specifically evaluate the contribution of the alternative pathway of NF-κB activation, *nfκb2* ^−/−^ BMDM [38], which lack both p100 and p52, and their C57BL/6 wild-type controls, were also used. When classical NF-κB activity was inhibited by overexpression of the super-repressor (SR), apoptosis occurred at 72 h p.i. later than in control WT J774 cells (Figure 6A, B), as in the case of overexpression of a constitutive negative mutant of IKK2 (IKK2 DN) (data not shown). In contrast, overexpression of a constitutive active mutant of IKK2 (IKK2 DA), which led to constitutively enhanced activation of NF-κB (Figure S6C), reversed the trend and resulted in advanced apoptosis at 24 h p.i.. Remarkably, no apoptosis was observed in the absence of the alternative pathway of NF-κB activation (*nfκb2* ^−/−^) (Figure 6B), nor when IKK1 DN stable J774 clones were used. This similar behaviour of *nfκb2* ^−/−^ BMDM and IKK1 DN stable J774 clones indicated a specific blockade of the alternative pathway in these stable clones, as inferred from their biochemical characterization showing constant p52 levels during fungal infection (Figure S6C, D). These results suggested that the alternative activation pathway of NF-κB was essential for pathogen-induced PCD, whereas the classical pathway controlled its onset. To unravel the molecular mechanisms controlled by NF-κB, which could account for fungal-induced cell apoptosis, we screened by Western blotting total protein extracts from WT or mutant J774 cells for activation of various apoptosis effectors (Figure S7). In WT J774 cells, selective proteolytic cleavage of apoptosis initiator caspases (caspase-8 and -9), executioner caspase (caspase-3) and poly(ADP-Ribose) polymerase (PARP), both a substrate of caspase-3 and a caspase-independent apoptosis effector, was observed upon *C. neoformans* infection. FASL and TRAIL-R1/DR4 protein levels were also increased in WT J774 from 24 h p.i. onward (Figure S8). Inhibition of classical NF-κB dimers delayed production of the above-mentioned activated caspases and PARP upon infection, whereas overexpression of a constitutive active mutant of IKK2 (IKK2 DA) led to earlier and stronger fungal-induced proteolytic processings. (Figure S7). When the IKK1-dependent pathway (IKK1 DN) was constitutively repressed, fungal infection triggered less active forms of caspases to levels that were below the threshold of effective DNA fragmentation (Figure S7 and Figure 6B). Altogether, these results indicated that fungal infection induced PCD in macrophage-like cells in an NF-κB-dependent manner through both the extrinsic (ligand-receptor linked) and intrinsic (mitochondrion-mediated) apoptosis activation pathways.

### Fungal alteration of macrophage cell cycle required a critical NF-κB dosage and involved the modification of expression of several cell cycle regulators in G0/G1, S and G2/M phases

**Figure 7:**
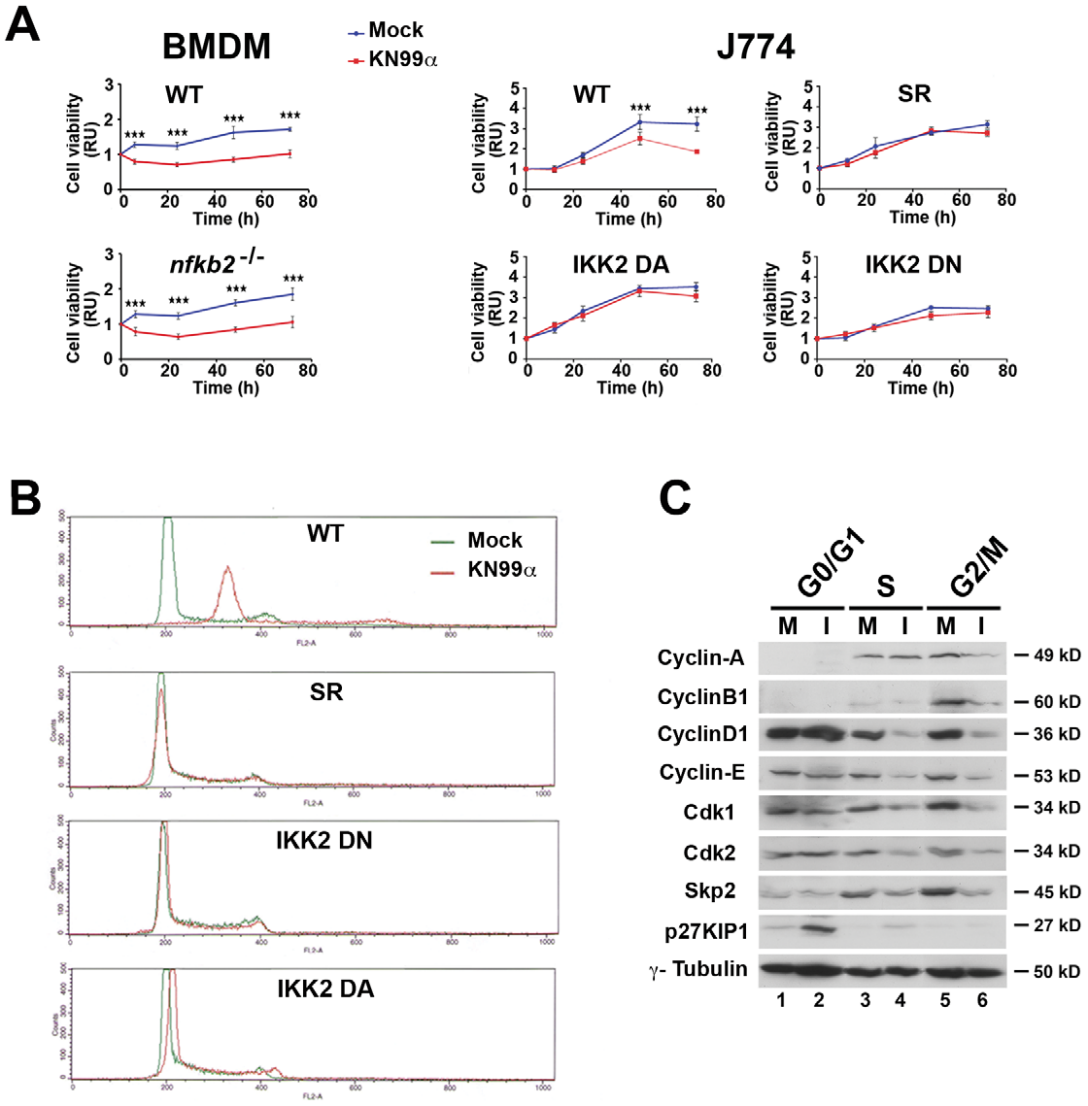
Fungal-alteration of cell cycle requires a critical NF-κB dosage and involves the modification of expression of several cell cycle regulators in G0/G1, S and G2/M phases. (**A**) Time-course of cell viability of mock-treated or *C. neoformans*-infected (KN99α) C57BL/6 control or *nfκb2* ^−/−^ BMDM or J774 cells, WT or stable NF-κB-modulated clones was assessed as mentioned previously. BMDM were cultured in presence of 30% CSF-conditioned medium from L929 cells and used directly without synchronization by M-CSF starvation. Results are presented as fold relative to the cell viability in mock-treated cells at time 0. Data are mean ± s.e.m. (BMDM n = 3, J774 n = 6). ***, *P*<0.001. (**B**) Representative flow cytometry histograms of cell cycle distribution of mock-treated or *C. neoformans*-infected (KN99α) WT J774 cells or stable NF-κB-modulated clones after 48 h, evaluated by propidium iodide incorporation (n = 6). (**C**) Representative western blot analysis of cyclins -D1, -E, -A, -B1, cyclin kinases, cdk1, cdk2 and Skp2, and cdk2 inhibitor, p27^KIP1^, expression levels in FACS-sorted viable cells stained with Hoechst at the different cell cycle phases. γ-tubulin was used as an internal loading control. 25 µg of total protein extracts were analyzed from mock-treated (M) or 48 h *C. neoformans* infected (KN99α) (I) WT J774 cells (n = 3).

We then investigated whether NF-κB regulated similarly fungal-induced inhibition of cell viability and alteration of cell cycle. In the absence of the alternative pathway of NF-κB activation (*nfκb2* ^−/−^), a significant decrease in viability of *nfκb2* ^−/−^ BMDM measured by ATPmetry was observed upon fungal infection, as in control C57BL/6 macrophages (Figure 7A). In these primary C57BL/6 macrophages, a very low contribution of apoptosis to cell viability (as fungal-induced apoptosis in WT BMDM reached 7% maximum of total cells (Figure 6B)) explained the similar cell viability curves of WT and mutant BMDM. Altogether, these data indicated that the alternative pathway was not required for fungal-induced cell viability inhibition. Remarkably, when the level of classical NF-κB activity was modified in whatever way, infected cells displayed no significant difference in viability compared to mock-treated cells in contrast to what was observed in infected WT J774 cells (Figure 7A). Consistently, no effect on cell cycle was seen either upon infection in any of these stable J774 clones with constitutively altered classical NF-κB activity (Figure 7B). When the alternative pathway of NF-κB activation (*nfκb2* ^−/−^) was abrogated, no modification of the number of mitotic cells compared to WT was detected at any time by phospho-histone H3 (Ser10) immunostaining (data not shown), confirming unaltered cell proliferation by *C. neoformans* in these mutant BMDMs devoid of p100 and p52. Therefore, only the classical pathway of NF-κB activation was indispensable for fungal-triggered inhibition of cell growth and alteration of cell cycle.

To decipher the molecular mechanisms by which *C. neoformans* repressed cell cycle progression, we purified by cell sorting mock treated (M) or 48 h-infected (I) J774 macrophages at various phases of the cell cycle and analyzed expression of an array of mitotic regulators by SDS PAGE and Western blotting (Figure 7C). Fungal infection led to enrichment of p27^KIP1^, a universal cyclin-dependent kinase inhibitor in G0/G1 and S phases. Conversely, cyclin-dependent kinases - such as Cdk2 (which drives transition from G1 to S phase by interacting with cyclin-A and -E) and Cdk1 (responsible together with cyclin-B1 for cell cycle progression from G2 to mitosis) as well as S-phase kinase-associated protein 2 (Skp2) - were down-regulated in both S and G2/M phases. Similarly, cyclin-D1 and -E levels diminished in S and G2/M, and cyclin-A and -B1 levels in G2/M phase. Thus, concerted regulation of cell cycle effectors was orchestrated by *C. neoformans* infection and promoted cell cycle impairment at the S and G2/M phases.

### Fungal-induced aneuploidy depended on NF-κB activity and involved modification of Mad2 expression levels

**Figure 8:**
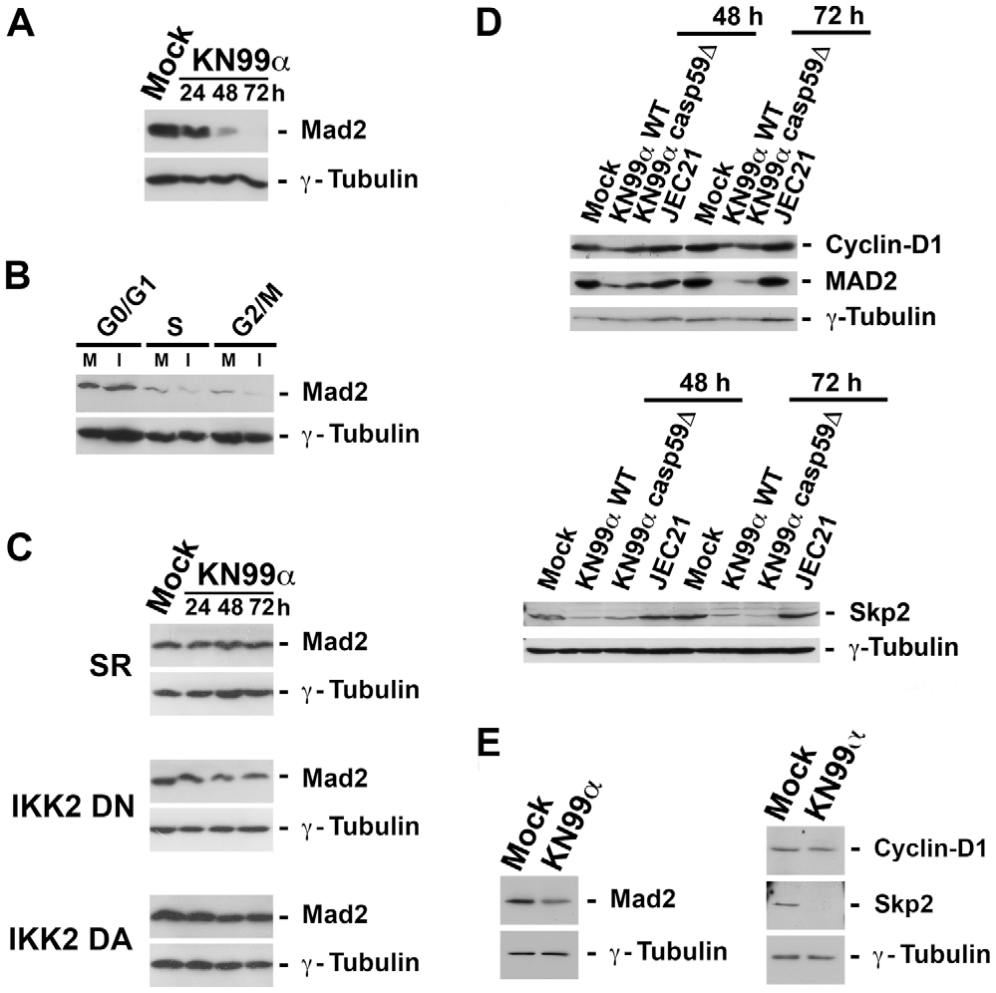
NF-κB-dependent reduction of Mad2 levels upon fungal infection. (**A**) Immunoblot analysis of the expression levels of Mad2 from total protein extracts (25 µg) of WT J774 mock-treated or infected by *C. neoformans* (KN99α) for the indicated time-points (n = 2). (**B**) Representative western blot analysis of Mad2 expression in FACS-sorted viable cells stained with Hoechst at the different cell cycle phases. 25 µg of total protein extracts were analyzed from mock-treated (M) or 48 h-infected KN99α *C. neoformans* (I) WT J774 cells (n = 3). (**C**) Immunoblot analysis of the expression levels of Mad2 from total protein extracts (25 µg) of stable J774 clones with impaired NF-κB activity/signalling (SR, IKK2 DN or IKK DA) mock-treated or infected by *C. neoformans* (KN99α) for the indicated time-points (n = 2). (**D**) Immunoblot analysis of the expression levels of Mad2, cyclin-D1 and Skp2 in total protein extracts (25 µg) from mock-treated J774 cells or from J774 cells infected with WT (KN99α) or acapsular mutant (*cap59*D) serotype A strains, or with serotype D strain (JEC21) at the indicated time-points (n = 2). (**E**) Immunoblot analysis of the expression levels of Mad2, cyclin-D1 and Skp2 in alveolar macrophage total protein extracts (25 µg) from mock-treated or KN99α *C. neoformans*-infected κB-*lacZ* mice 3 d p.i.. (n = 3). γ-tubulin was used as an internal loading control.

As DNA damage may cause chromosomal instability, we then asked whether the number of γ-H2AX foci, markers of DNA double-strand breaks, were modified by fungal infection. The absence of significant increase in γ-H2AX foci upon *C. neoformans* infection (Figure S9) and lack of detection of p53 or phosphorylated CHK1 in J774 macrophage-like cells (data not shown) argued against fungal activation of the DNA damage signalling cascade. To search for the molecular mechanisms responsible for fungal-triggered aneuploidy, we next looked for putative modification levels upon *C. neoformans* infection of proteins that have been demonstrated to drive chromosome missegregation and instability when overexpressed or down-regulated, such as Mad2 a protein essential for spindle assembly during mitosis [39], [40]. Indeed, fungal infection elicited a strong decrease in total Mad2 levels from 48 h p.i. onward (Figure 8A) and more specifically in the S and G2/M phases of cell cycle (Figure 8B). This suggested that fungal-induced aneuploidy was mediated at least partly by Mad2 down-regulation levels. The observation upon infection of a normal cell cycle and DNA content in J774 stable clones with impaired NF-κB activity or signalling (Figure 6B) suggested that these processes were regulated by NF-κB. Consistently, reduced levels of cyclin-D1 upon *C. neoformans* infection were observed in WT J774 cells only but not in these stable J774 clones (data not shown). We then asked whether modifications of Mad2 levels upon fungal infection depended on NF-κB activation. Western blot of total protein extracts from stable J774 clones with impaired NF-κB activity or IKK2-dependent signalling revealed that fungal-induced changes in Mad2 levels disappeared when classical NF-κB activity or signalling was inhibited or increased (Figure 8C). Therefore, fungal-induced aneuploidy was most likely the consequence of NF-κB-controlled Mad2 down-regulation. As for WT (KN99α) *C. neoformans*, the acapsular strain (*cap59*D), which also induced cell cycle alteration and aneuploidy (Figure 4B, C), led upon infection to decreased levels of MAD2, cyclin-D1 and Skp2 (Figure 8D), in contrast to serotype D strain (JEC21), which had no impact on the levels of these proteins as expected. To determine whether fungal-induced effects on cell cycle and aneuploidy applied to tissue macrophages *in vivo*, we next isolated alveolar macrophages from κB-*lacZ* reporter transgenic mice mock-treated or infected for 3 d with WT (KN99α) *C. neoformans*. Western blot analysis of total protein extracts revealed that although cyclin-D1 levels were unaffected, both MAD2 and Skp2 levels were diminished in alveolar macrophages upon *in vivo* infection (Figure 8E). Thus even in tissue macrophages *in vivo*, down-regulation of MAD2 and a cell cycle control protein such as Skp2, both regulated by NF-κB, were triggered by a fungal pathogen.

## Discussion

Regulation of host cell survival by pathogens has emerged as a way to control progression of innate immune responses upon infection. In this study, we report that *C. neoformans* directly affected two host functions essential for macrophage viability, apoptosis and cell cycle, in an NF-κB-dependent manner and describe for the first time induction of aneuploidy by a fungal pathogen.

We show that in macrophages *C. neoformans* induced both the classical and alternative NF-κB activation pathways. Analyses of mice harbouring a knock-in of a IKK1-kinase dead mutant at the *ikka* locus [29] or mice with myeloid-specific IKK2 knock-out have shed light on a peculiar function of IKK1-dependent signalling as well as on myeloid-specific IKK2-dependent signalling [41] in the suppression of M1 macrophage activation. Notably, increase in nuclear p50/p50 dimers, previously described to direct macrophages towards a M2 anti-inflammatory phenotype [42], was also elicited by this fungal pathogen in macrophage-like cells J774 (Figure 4B lanes 1, 2). Hence, rise in nuclear pools of p50 homodimers, p50- and p52-heterodimers may in part explain how NF-κB contributes to the immune tolerance triggered by unopsonised *C. neoformans*. Importantly, analysis of infected κB-*lacZ* reporter transgenic mice in experimental conditions that mimicked the systemic infection in humans indicated that such activation of NF-κB by *C. neoformans* occurs *in vivo*.

Fungal infection compromised macrophage viability in two ways. First, as other microbes [43] *C. neoformans* induced macrophage apoptosis. Programmed cell death of inflammatory cells is one of the physiological mechanisms that contributes to the resolution of inflammation [44] since their apoptosis decreases tissue damage and limits the inflammatory response. Use of WT as well as mutant strains of *C. neoformans* disclosed an essential PCD-promoting role of the capsule, in accordance with previous reports obtained with purified *C. neoformans* capsular polysaccharides in T cells or macrophages [21]–[24], [45]. Fungal-induced PCD might thus result from direct interaction of the fungus with the macrophage, although recognition of unopsonised *C. neoformans* by macrophages is usually poor, or from isolated capsule, as capsule shedding commonly occurs during cryptococcal infections [8]. Analysis of stable clones with suppressed NF-κB activity or impaired IKK1- or IKK2-dependent signalling, or primary BMDM without alternative NF-κB activation revealed that fungal-induced apoptosis required differentially these activation pathways upon infection. The alternative pathway and IKK1-dependent signalling were essential, while the classical pathway of NF-κB activation and IKK2-dependent signalling controlled its onset through activation of caspase-8, -3, -9 and PARP cleavage. NF-κB can behave as a cell death promoting or protecting factor depending on the nature of stimulus and cell type involved, which together determine cell fate [46]. As such, IKK1-dependent signalling is essential for group B *Streptococcus*-induced apoptosis of macrophages, as it is here for a fungal pathogen [29]. Proapoptotic activity is thought to proceed through some of NF-κB transcriptional targets, including FAS and its ligand or TRAIL receptors and TRAIL [47]. Consistently, upregulation of FASL and TRAIL-R1/DR4 receptor was detected in J774 cells 24 h p.i..

Targeting host cell multiplication through modulation of the cell cycle is another survival scheme commonly followed by bacteria and viruses [48], [49]. We report here that *C. neoformans* significantly affected macrophage viability, proliferation and cell cycle progression. It is noteworthy to point out that in our experimental settings for the various cell types used (macrophagic-like J774 cells, BALB/c or C57BL/6 BMDM), macrophage viability mainly reflected cell growth, as fungal-induced apoptosis remained low (around or below 10%), except for BALB/c BMDM where it reached up to 27% 72 h p.i.. GXM-, O-acetylation- or GalXM-deficient strains behaved as WT serotype A *C. neoformans* strain. These results indicated that inhibition of macrophage viability occurred in a capsule-independent fashion. They differ from the inhibition of human T cell proliferation reported by purified GalXM, as the latter might display induction of apoptosis, an effect induced by the high concentrations of polysaccharide used in these experiments that are not reached in our experimental settings when using the whole yeast [21]. The fact that serotype D *C. neoformans* strain had no significant impact on cell viability at M.O.I. 5 (data not shown) and led to a mild effect even at high M.O.I. (20), with a viability curve which paralleled that of mock-treated cells, suggests that here *C. neoformans*-induced inhibition of viability at high M.O.I. most probably reflected an increase of apoptosis. Inhibition of proliferation per se is therefore a likely specific feature of *C. neoformans* var. *grubii* (serotype A) strain, which diverged 18 million years ago from *C. neoformans* var. *neoformans* (serotype D) strain [50]. It further demonstrates that this effect, which we have shown to require both cell contact and viable yeasts, is not due to a general stress response, but is genuinely induced by this fungus. Moreover we have established that analogous cell cycle alterations as well as mitotic regulators modifications were produced by WT and acapsular serotype A strains. Collectively these findings suggest that, as regards the pathogen side, the mechanism involved in the impairment of cell cycle and aneuploidy in macrophages is likely complex and will probably involve several fungal components. Consistent with our *in vitro* findings, inhibition of cell growth triggered by *C. neoformans* occurred *in vivo* in the whole spleen of κB-*lacZ* transgenics but surprisingly was not restricted to macrophages. This general cell growth inhibition by *C. neoformans in vivo* might thus result from other and yet undetermined mechanisms. *C. neoformans* compromised macrophage cell growth through perturbation of the cell cycle. Remarkably, phagocytosis of *C. neoformans* by macrophages was reported to transiently stimulate progression from G1 to S in both macrophage-like cells and primary BMDM with a concomitant increase of cyclin-D1 expression levels [51], [52]. However, when inside the macrophages, phagocytosed live yeasts suppressed BMDM growth by decreasing cyclin-D1 expression [52]. Thus according to its presence in the milieu as an extracellular or intracellular pathogen and to its survival strategy, *C. neoformans* modulates macrophage cell cycle for its own profit. Combined regulation of various cyclins and their corresponding kinases or kinase inhibitor was orchestrated by *C. neoformans* infection and promoted cell cycle impairment at the S and G2/M phases in J774 cells with a requirement for NF-κB distinct to that of PCD. Here, the alternative pathway of NF-κB activation was dispensable, whereas the classical pathway and the IKK2-dependent signalling were essential. Reduction or rise of classical NF-κB dimers similarly prevented fungal-induced inhibition of cell viability and alteration of the cell cycle. A critical NF-κB dosage seems therefore to be required for fungal-triggered perturbation of cell cycle and viability. In another context, survival of lymphocyte progenitors was also shown to rely upon a limited range of NF-κB activity [53]. Several cell cycle checkpoint effectors have been identified as NF-κB target genes, including cyclin-D1 and Skp2 [54], [55]. NF-κB regulation of the cell cycle is complex. It has been shown to control the G1/S transition of the cell cycle and to be required also for G2/M progression [56]. P52 or p50 heterodimers with RelB or c-Rel were associated with decreased expression of cyclin-D1 and Skp2 in S and G2 phases. Enforced expression of c-Rel causes growth arrest at the G1/S transition [57] and p65 down-regulates cyclin-E gene expression [58]. The induction of p52- and p65-heterodimers (Figure 4B) by fungal infection could therefore explain in part the down-regulation of cyclin-D1, -E and Skp2. Interestingly, when analysing the behaviour of tissue macrophages upon *in vivo* infection by *C. neoformans*, only reduction of Skp2 levels were observed in alveolar macrophages. The absence of cyclin-D1 variation is most likely due to the limited proliferative potential of these cells. Anyway, it remains that the significant decrease of Skp2 levels in tissue macrophages, both an NF-κB target and an important cell cycle regulator, argues strongly in favour of the *in vivo* relevance of this cytotoxicity mechanism.

Modification of host cell ploidy through perturbation of cell cycle checkpoints is often triggered by viruses, including HIV and HCV [59], [60], and bacteria, such as *H. pylori* or E. *faecalis* [61]. Our results display the first report of induction of aneuploidy by a fungal pathogen. All observations have been made on both macrophagic-like cell line and primary cells (BMDM) indicating that our data cannot be attributed to specificities of cancer or immortalized cells, which are prone to become aneuploid. Moreover, the absence of a significant increase in γ-H2AX foci upon *C. neoformans* infection of macrophages argues against fungal activation of the DNA damage signalling cascade. It also suggests that mammalian DNA integrity is not directly affected by *C. neoformans* infection. Decreased levels of the spindle assembly checkpoint protein Mad2 were observed in whole cell extracts or cell-sorted infected macrophages at the S and G2/M phases. Genetic analyses of both human cancer cells and murine primary embryonic fibroblasts harbouring only one allele of Mad2 have correlated Mad2 haplo-insufficiency to defective mitotic checkpoint, elevated rate of chromosome missegregation and aneuploidy [39]. Therefore, it is likely that fungal-induced aneuploidy may result from Mad2 down-regulation. Moreover, this aneuploidy depends on NF-κB activation since fungal-induced modification of DNA content and changes in Mad2 levels disappeared in stable J774 clones with modulated NF-κB activity or signalling. Importantly, analyses of alveolar macrophages from mock-treated or *C. neoformans*-infected mice revealed a significant decrease of MAD2 levels upon infection, suggesting that aneuploidy may also be triggered *in vivo* by this fungus.

Together, these findings provide novel insight into our understanding of the mechanisms whereby a fungal pathogen hijacks and shapes the host immune response to its own benefit through in part uncoupling of NF-κB activity in apoptosis, and cell cycle impairment and aneuploidy. These findings may have also wider implications as studies with mouse models of chromosome instability [62] have shown that aneuploidy may directly contribute to tumour formation. More specifically Mad2 haplo-insufficiency leads to high frequency of lung carcinoma [39] and carcinogenesis is enhanced when p53 is absent [63]. In certain contexts and when oncogenic or tumour-suppressor loci are mutated, fungal infection might therefore potentially participate via aneuploidy induction to tumourigenesis.

## Materials and Methods

### Ethics statement

This study was carried out in strict accordance with the French and European regulations on care and protection of the Laboratory Animals (EC Directive 86/609, French Law 2001-486 issued on June 6, 2001). Animals were housed in the Institut Pasteur animal facilities accredited by the French Ministry of Agriculture to perform experiments on live mice (accreditations # A 75 15-27 and B 75 15-05 issued on November 12, 2004 and May 22, 2008 respectively). The protocol was approved by the veterinary staff of the Institut Pasteur animal facility and was performed in compliance with the NIH Animal Welfare Insurance #A5476-01 issued on 02/07/2007. All efforts were made to minimize suffering during animal handling and experimentation.

### Cells, mice and infection

Murine macrophage cells, construction of J774 stable clones with NF-κB-modulated activity or signalling, transgenic mice and infection conditions are described in Protocol S1.

### *C. neoformans* strains

Wild-type and mutant *C. neoformans* var. *grubii* (serotype A) strains, *C. neoformans* var. *neoformans* (serotype D) strain and growth conditions are described in Protocol S1.

### Reichert differential interference contrast (DIC) imaging, apoptosis and viability assays

Reichert DIC imaging, apoptosis and viability assays were done as described in Protocol S1.

### Immunohistocytochemistry

Cultured cells or spleen tissue sections were processed for immunocytochemistry as described in Protocol S1.

### Cell cycle analysis, cell sorting and karyotyping

Details of cell cycle analysis by flow cytometry, karyotype obtention and sorting of J774 cells at the various phases of the cell cycle are provided in Protocol S1.

### Electrophoretic mobility shift assay (EMSA), reporter assay and Western blotting

EMSA, NF-κB-luciferase assay and immunoblot analysis were performed as described in Protocol S1.

### Statistics

Statistical analysis was done as described in Protocol S1.

## Supporting Information

Figure S1 **Absence of significant phagocytosis by J774 cells with unopsonised** ***C. neoformans*** **.** (**A**) Reichert differential interference contrast (DIC) images of J774 cells mock-treated or infected by WT (KN99α) or acapsular mutant (*cap59*D) *C. neoformans* 48 h p.i. Scale bar 10 µm. (**B**) Quantification of the number of phagocytic cells per total number of J774 cells, mock-treated or *C. neoformans*-infected by WT (KN99α) or acapsular mutant (*cap59*D) strains, at the indicated time-points. Data are mean ± s.e.m. (counted cells; n = 500). **, *P*<0.01. ***, *P*<0.001.
(TIF)

Figure S2 **Fungal-induced inhibition of cell viability is independent of GalXM.** Time-course of cell viability of J774 cells mock-treated or infected by WT (KN99α) or capsule mutant *C. neoformans* strains devoid of GalXM (2 independent suppressors of *uge1*D mutant (*uge1*D supp1, *uge1*D supp4) with a doubling time similar to that of WT at 37°C), was assessed using a commercial viability assay generating a luminescent signal directly proportional to the amount of ATP present in metabolically active cells. Results are presented as fold relative to the cell viability in mock-treated cells at time 0. Data are mean ± s.e.m. (n = 6). ***, *P*<0.001, compared with the mock-treated cells. M.O.I. was 5 for WT strain and 10 for GalXM mutants.
(TIF)

Figure S3 **Fungal-induced inhibition of cell viability requires viable yeasts and pathogen-cell contact.** (**A**) Time-course of cell viability of J774 cells mock-treated or infected by intact or heat-inactivated WT *C. neoformans* (KN99α) was assessed using a commercial viability assay generating a luminescent signal directly proportional to the amount of ATP present in metabolically active cells. Results are presented as fold relative to the cell viability in mock-treated cells at time 0. Data are mean ± s.e.m. (n = 6). ***, *P*<0.001, compared with mock-treated cells or with heat-inactivated KN99α-infected cells. (**B**) Time-course of cell viability of J774 cells mock-treated or infected by WT *C. neoformans* (KN99α) was assessed as mentioned above after separate incubation of cells and yeasts in a two chambers system (transwell). Data are mean ± s.e.m. (n = 6).
(TIF)

Figure S4 **Fungal-induced disruption of cell cycle also occurs in primary macrophages.** (**A**) Representative flow cytometry histograms of cell cycle distribution of mock-treated or *C. neoformans*-infected (KN99α) J774 cells after 72 h, assessed by propidium iodide incorporation (n = 6). (**B**) Representative flow cytometry histograms of cell cycle distribution of mock-treated or *C. neoformans*-infected (KN99α) BMDM after 48 h (upper panel) or 72 h (lower panel) assessed by propidium iodide incorporation (n = 2). BMDM were cultured in presence of 30% CSF-conditioned medium from L929 cells and used directly without synchronization by M-CSF starvation. Arrow indicates an increase in DNA content (**A**, **B**). 30000 total events were acquired per sample with identical parameters of acquisition for all samples and data were analysed as described in Protocol S1.
(TIF)

Figure S5 **Confirmation of fungal-induced disruption of cell cycle by invariance of chicken red blood cells standard peak.** (**A**) Representative flow cytometry histograms of cell cycle distribution of mock-treated or *C. neoformans*-infected (KN99α) J774 cells after 48 h, assessed by propidium iodide incorporation. (**B**) Representative flow cytometry histograms of cell cycle distribution of samples shown in (**A**), assessed by propidium iodide incorporation in the presence of chicken red blood cells (CRBC). X-axis shows intensity of fluorescence and Y-axis number of cells. 30000 total events were acquired per sample with identical parameters of acquisition for all samples and data were analysed as described in Protocol S1. (n = 2). Arrow indicates an increase in DNA content (in **A** & **B**). Arrowhead points the invariable CRBC specific peak (in **B**).
(TIF)

Figure S6 **Characterisation of stable J774 clones with modulated NF-κB activity.** (**A**) Scheme presenting the various IKK mutants or super-repressor used to generate constitutive inhibition (SR or IKK2 DN) or activation (IKK2 DA) of the IKK2-dependent pathway of NF-κB activation or inhibition of the IKK1-dependent pathway (IKK1 DN) in J774 macrophages. (**B**) Western analysis of total protein extracts (20 µg) from several independent clones (1, 2 or 1, 2, 3) for each mutant revealing the constitutive expression of each specific mutant protein. -tubulin is used as an internal loading control. (**C**) EMSA analysis of total protein extracts (30 µg) from several independent clones (1, 2 or 1, 2, 3) for each mutant demonstrating specific activation or inhibition of NF-κB. In IKK2 DA mutant clones classical NF-κB dimers increase drastically, whereas they strongly diminish in IKK2 DN mutant or SR mutant clones. In IKK1 DN clones, there is no or very slight effect on classical NF-κB dimers whereas a complete disappearance of p52-containing alternative dimers is observed. (**D**) Representative western analysis of total protein extracts (25 µg) from one IKK1 DN clone, mock-treated or infected for the indicated times by wild-type (KN99α) *C. neoformans*, with antibodies against p100 (or γ-tubulin as an internal loading control), showing suppression of the p100 processing in p52 and consequently unvarying p52 levels upon fungal infection.
(TIF)

Figure S7 **Fungal infection promotes induction of the extrinsic and intrinsic apoptosis activation pathways in an NF-κB-dependent manner.** Immunoblot analysis of the expression levels of cleaved caspase-8 (18 and/or 43 kD), caspase-3 (17 kD), caspase-9 (37 kD), PARP (89 kD), as well as -tubulin (internal control) from total protein extracts (20 mg) of WT or stable mutant J774 cells mock-treated or infected by *C. neoformans* (KN99α) for the indicated time-points (n = 3). Arrowhead points out immunogenic specific band.
(TIF)

Figure S8 **Fungal infection promotes FASL and TRAIL-R1/DR4 increase.** Immunoblot analysis of the expression levels of FASL and TRAIL-R1/DR4, as well as γ-tubulin (internal control) from total protein extracts (30 mg) of J774 cells mock-treated or infected by *C. neoformans* (KN99α) for the indicated time-points (n = 2).
(TIF)

Figure S9 **Absence of significant increase in γ-H2AX foci upon fungal infection.** (**A**) Phospho-H2AX (p-H2AX or γ-H2AX) immunofluorescence and DAPI staining of mock-treated or *C. neoformans*-infected (KN99α) J774 cells 48 h p.i.. Scale bar 20 mm. (**B**) P-H2AX immunofluorescence and DAPI staining of mock-treated or *C. neoformans*-infected (KN99α) BMDM 48 h p.i.. Scale bar 20 µm. (**C**) Quantification of the number of p-H2AX foci per total number of cell nuclei (blue) in mock-treated or *C. neoformans*-infected (KN99α) J774 cells at the indicated time-points. Data are mean ± s.e.m. (nuclei; n = 300). (**D**) Quantification of the number of Phospho-H3^+^ nuclei per total number of cell nuclei (blue) in mock-treated or *C. neoformans*-infected (KN99α) BMDM at the indicated time-points. Data are mean ± s.e.m. (nuclei; n = 325).
(TIF)

Protocol S1 **Detailed Material and Methods used in this study.** Cell culture and infection, Mice and infection, *C. neoformans* strains, Reichert differential interference contrast (DIC) imaging, Viability assay, Apoptosis assay, Immunohistochemistry, Cell cycle analysis by flow cytometry, Karyotyping cells, Electrophoretic mobility shift assay (EMSA), Reporter assay, Western blotting, Construction of J774 stable clones, Cell cycle sorting & Statistics.
(DOC)
