# Supplementary figures and images for: Fungal-Induced Cell Cycle Impairment, Chromosome Instability and Apoptosis via Differential Activation of NF-κB
Source: PLoS Pathog. 2012 Mar 1;8(3):e1002555. doi: 10.1371/journal.ppat.1002555 (PMC3291658; doi:10.1371/journal.ppat.1002555)

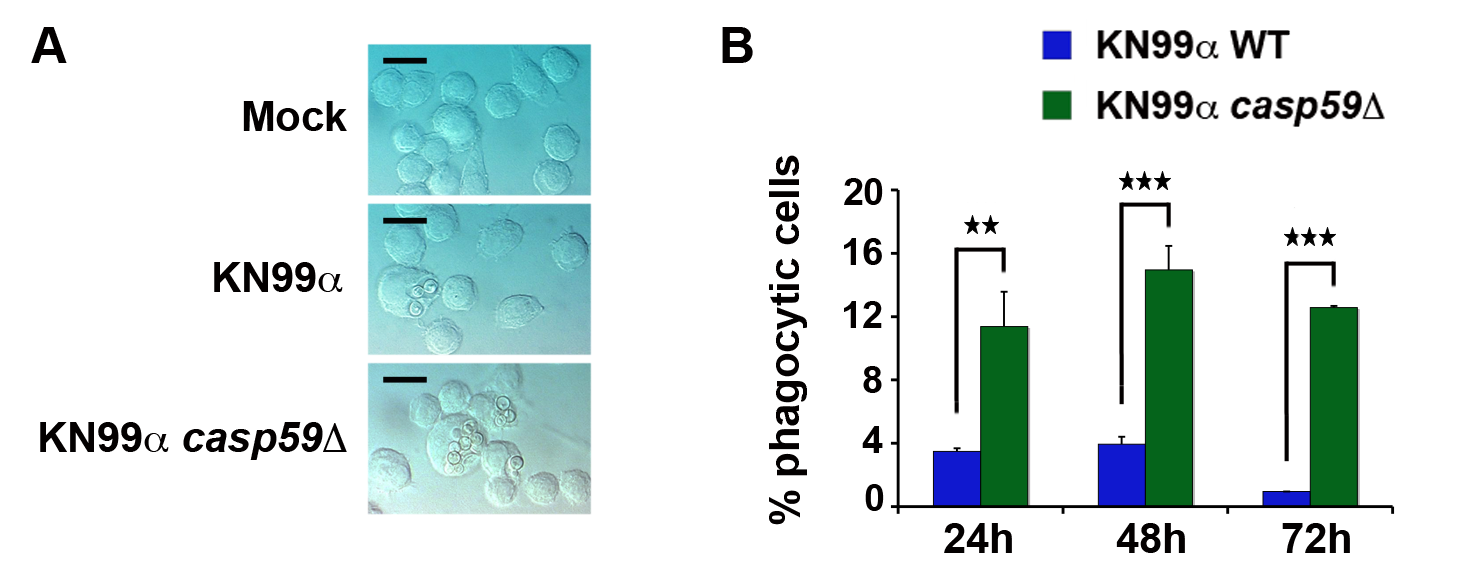

Supplement: Figure S1 — Absence of significant phagocytosis by J774 cells with unopsonised C. neoformans . (A) Reichert differential interference contrast (DIC) images of J774 cells mock-treated or infected by WT (KN99α) or acapsular mutant (cap59D) C. neoformans 48 h p.i. Scale bar 10 µm. (B) Quantification of the number of phagocytic cells per total number of J774 cells, mock-treated or C. neoformans-infected by WT (KN99α) or acapsular mutant (cap59D) strains, at the indicated time-points. Data are mean ± s.e.m. (counted cells; n = 500). **, P<0.01. ***, P<0.001. (TIF) [file ppat.1002555.s001.tif]

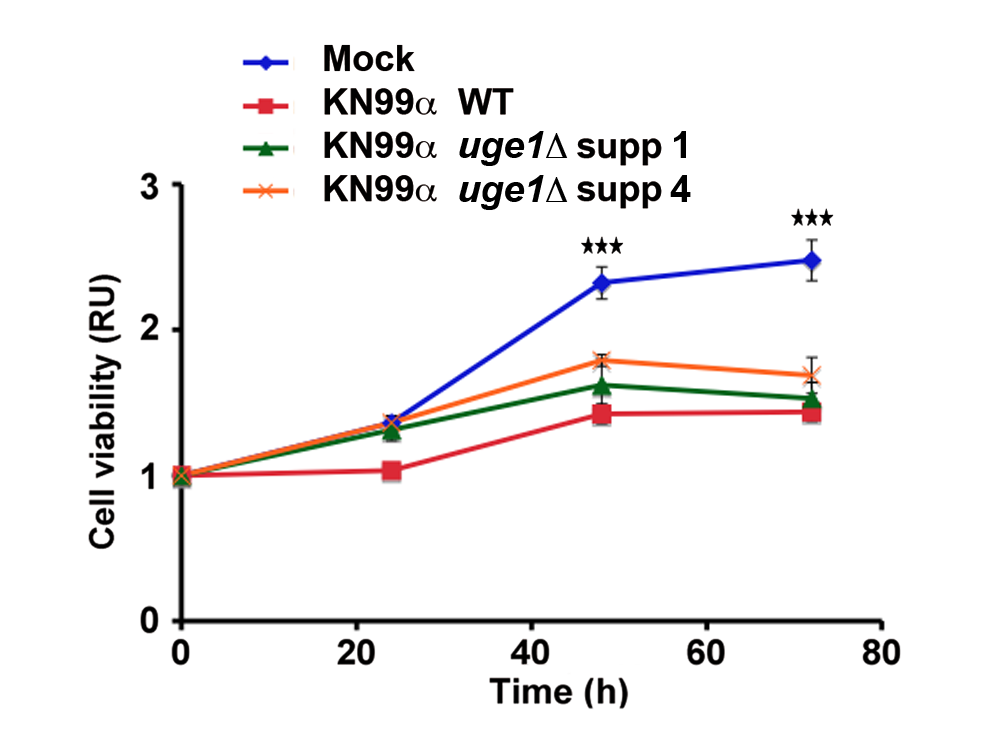

Supplement: Figure S2 — Fungal-induced inhibition of cell viability is independent of GalXM. Time-course of cell viability of J774 cells mock-treated or infected by WT (KN99α) or capsule mutant C. neoformans strains devoid of GalXM (2 independent suppressors of uge1D mutant (uge1D supp1, uge1D supp4) with a doubling time similar to that of WT at 37°C), was assessed using a commercial viability assay generating a luminescent signal directly proportional to the amount of ATP present in metabolically active cells. Results are presented as fold relative to the cell viability in mock-treated cells at time 0. Data are mean ± s.e.m. (n = 6). ***, P<0.001, compared with the mock-treated cells. M.O.I. was 5 for WT strain and 10 for GalXM mutants. (TIF) [file ppat.1002555.s002.tif]

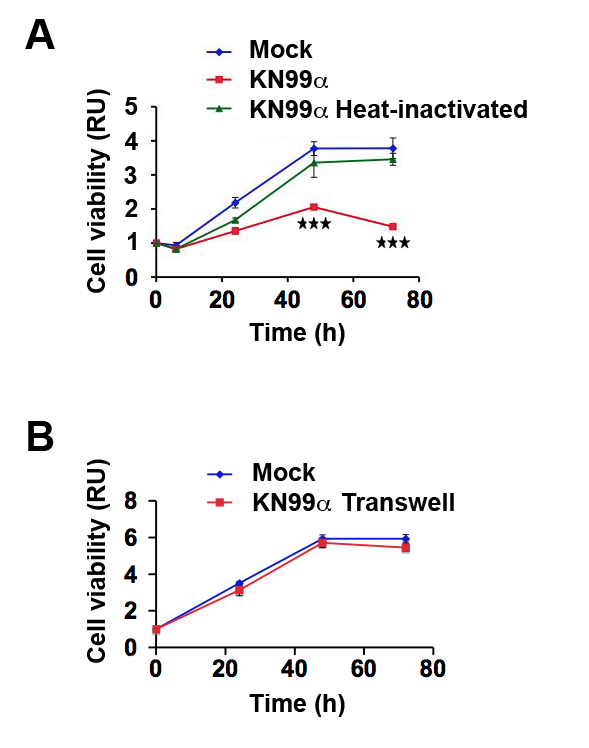

Supplement: Figure S3 — Fungal-induced inhibition of cell viability requires viable yeasts and pathogen-cell contact. (A) Time-course of cell viability of J774 cells mock-treated or infected by intact or heat-inactivated WT C. neoformans (KN99α) was assessed using a commercial viability assay generating a luminescent signal directly proportional to the amount of ATP present in metabolically active cells. Results are presented as fold relative to the cell viability in mock-treated cells at time 0. Data are mean ± s.e.m. (n = 6). ***, P<0.001, compared with mock-treated cells or with heat-inactivated KN99α-infected cells. (B) Time-course of cell viability of J774 cells mock-treated or infected by WT C. neoformans (KN99α) was assessed as mentioned above after separate incubation of cells and yeasts in a two chambers system (transwell). Data are mean ± s.e.m. (n = 6). (TIF) [file ppat.1002555.s003.tif]

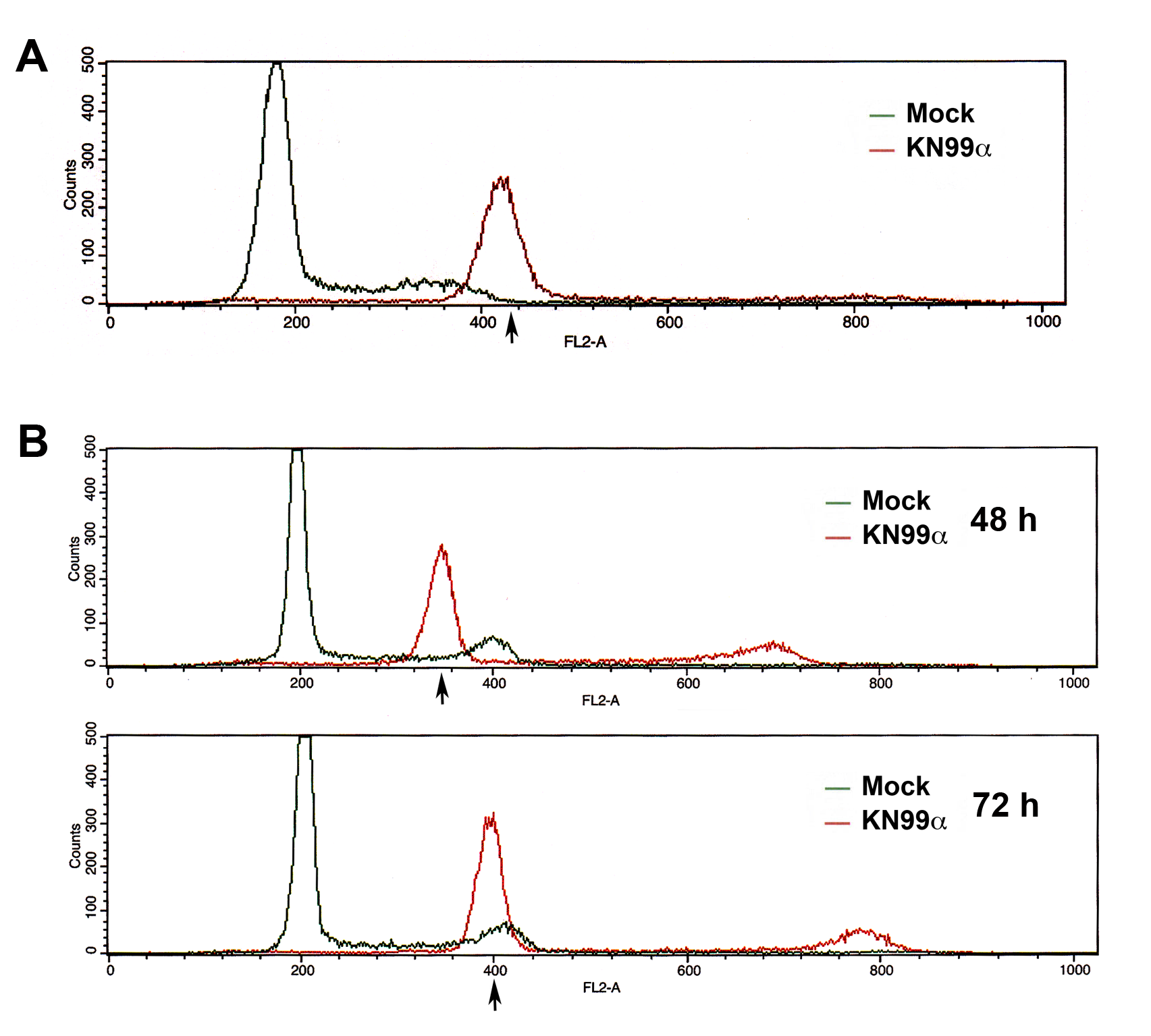

Supplement: Figure S4 — Fungal-induced disruption of cell cycle also occurs in primary macrophages. (A) Representative flow cytometry histograms of cell cycle distribution of mock-treated or C. neoformans-infected (KN99α) J774 cells after 72 h, assessed by propidium iodide incorporation (n = 6). (B) Representative flow cytometry histograms of cell cycle distribution of mock-treated or C. neoformans-infected (KN99α) BMDM after 48 h (upper panel) or 72 h (lower panel) assessed by propidium iodide incorporation (n = 2). BMDM were cultured in presence of 30% CSF-conditioned medium from L929 cells and used directly without synchronization by M-CSF starvation. Arrow indicates an increase in DNA content (A, B). 30000 total events were acquired per sample with identical parameters of acquisition for all samples and data were analysed as described in Protocol S1. (TIF) [file ppat.1002555.s004.tif]

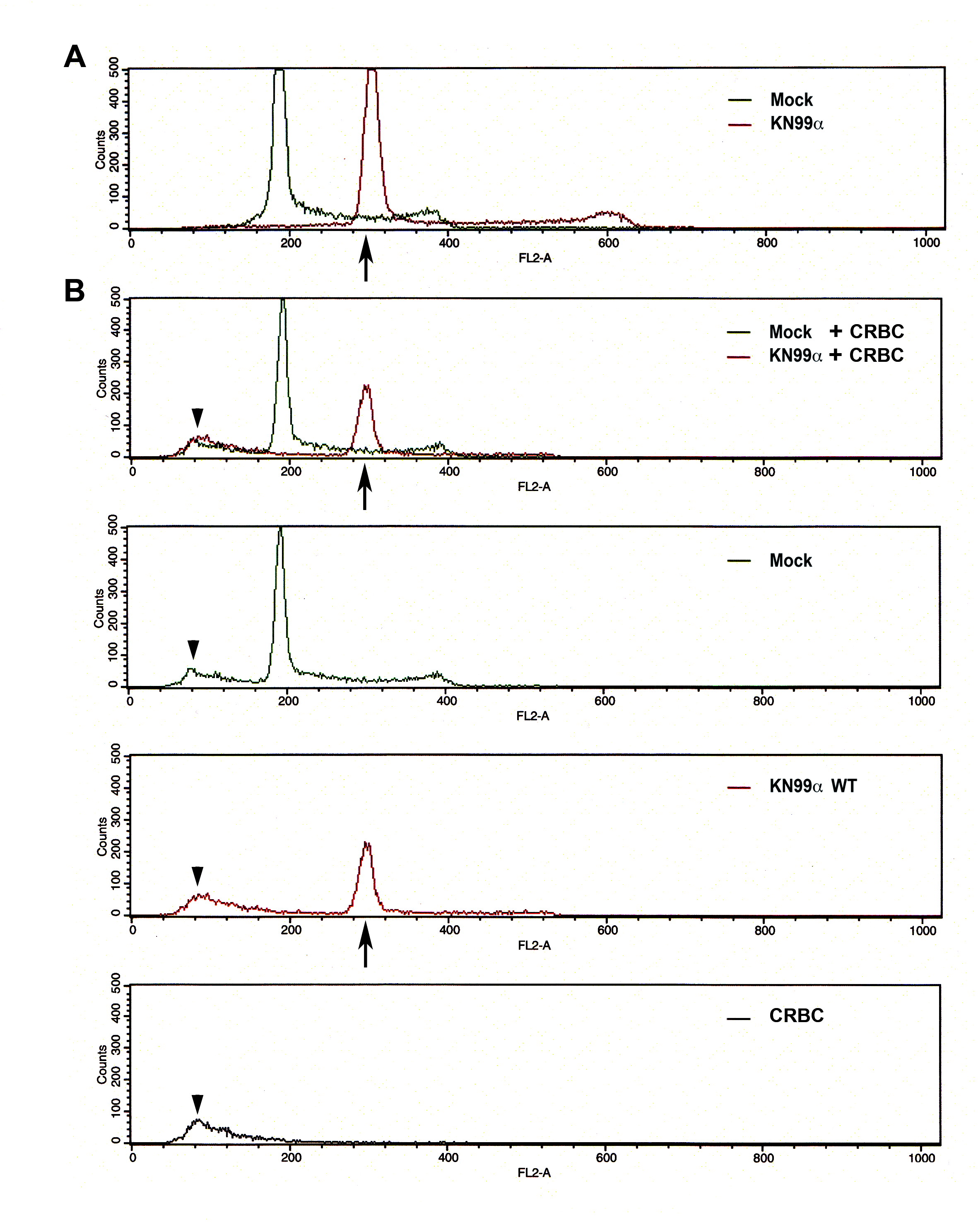

Supplement: Figure S5 — Confirmation of fungal-induced disruption of cell cycle by invariance of chicken red blood cells standard peak. (A) Representative flow cytometry histograms of cell cycle distribution of mock-treated or C. neoformans-infected (KN99α) J774 cells after 48 h, assessed by propidium iodide incorporation. (B) Representative flow cytometry histograms of cell cycle distribution of samples shown in (A), assessed by propidium iodide incorporation in the presence of chicken red blood cells (CRBC). X-axis shows intensity of fluorescence and Y-axis number of cells. 30000 total events were acquired per sample with identical parameters of acquisition for all samples and data were analysed as described in Protocol S1. (n = 2). Arrow indicates an increase in DNA content (in A & B). Arrowhead points the invariable CRBC specific peak (in B). (TIF) [file ppat.1002555.s005.tif]

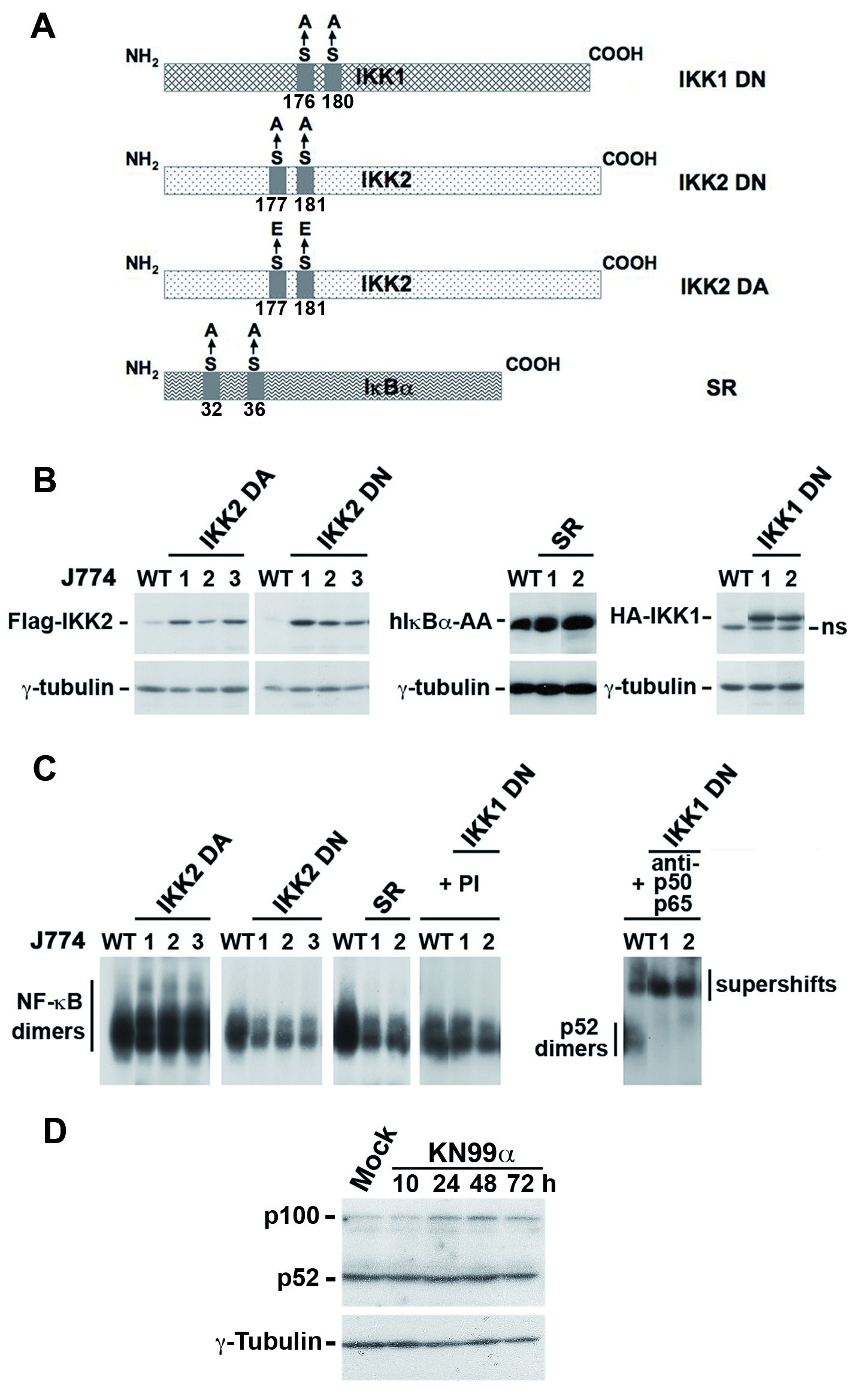

Supplement: Figure S6 — Characterisation of stable J774 clones with modulated NF-κB activity. (A) Scheme presenting the various IKK mutants or super-repressor used to generate constitutive inhibition (SR or IKK2 DN) or activation (IKK2 DA) of the IKK2-dependent pathway of NF-κB activation or inhibition of the IKK1-dependent pathway (IKK1 DN) in J774 macrophages. (B) Western analysis of total protein extracts (20 µg) from several independent clones (1, 2 or 1, 2, 3) for each mutant revealing the constitutive expression of each specific mutant protein. -tubulin is used as an internal loading control. (C) EMSA analysis of total protein extracts (30 µg) from several independent clones (1, 2 or 1, 2, 3) for each mutant demonstrating specific activation or inhibition of NF-κB. In IKK2 DA mutant clones classical NF-κB dimers increase drastically, whereas they strongly diminish in IKK2 DN mutant or SR mutant clones. In IKK1 DN clones, there is no or very slight effect on classical NF-κB dimers whereas a complete disappearance of p52-containing alternative dimers is observed. (D) Representative western analysis of total protein extracts (25 µg) from one IKK1 DN clone, mock-treated or infected for the indicated times by wild-type (KN99α) C. neoformans, with antibodies against p100 (or γ-tubulin as an internal loading control), showing suppression of the p100 processing in p52 and consequently unvarying p52 levels upon fungal infection. (TIF) [file ppat.1002555.s006.tif]

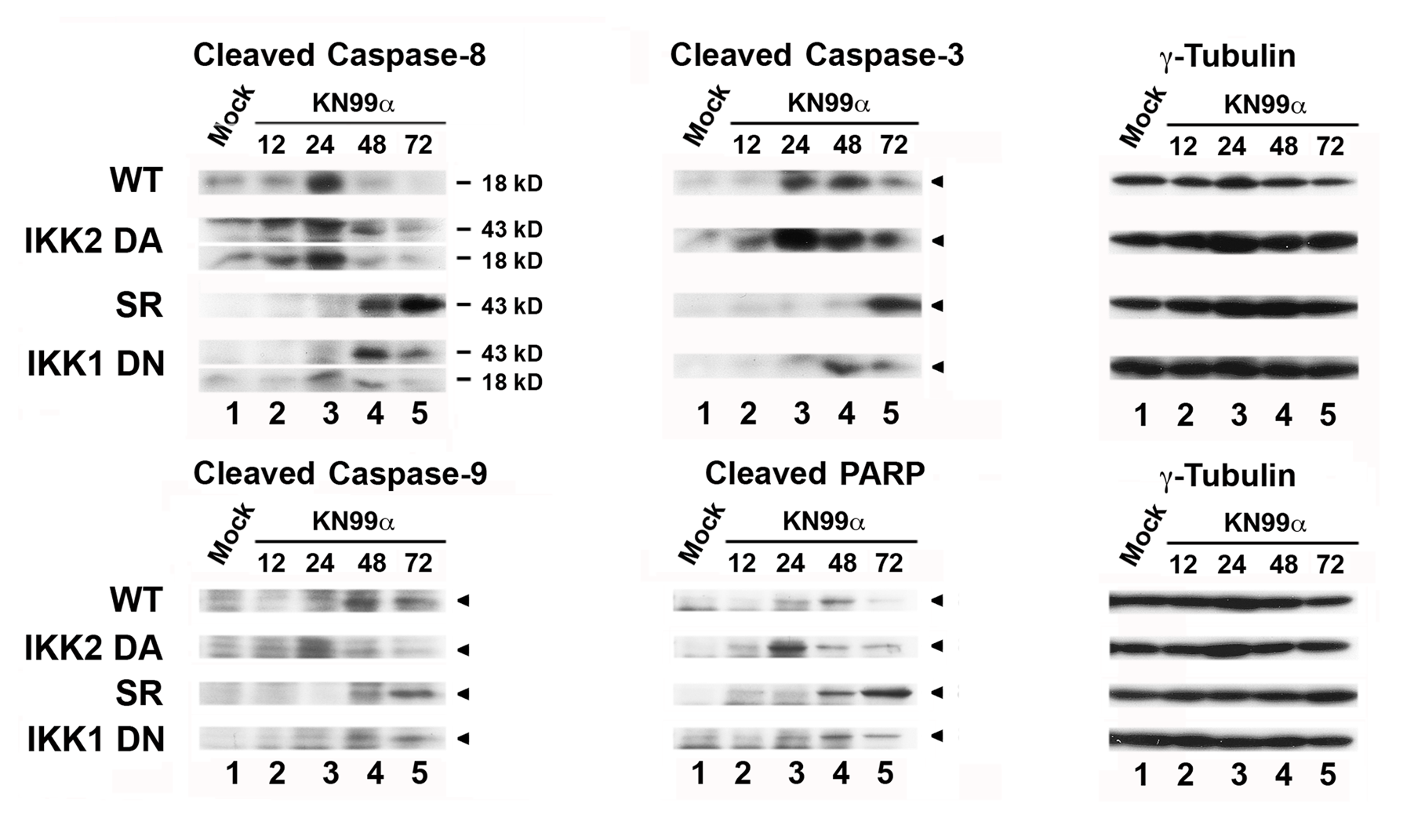

Supplement: Figure S7 — Fungal infection promotes induction of the extrinsic and intrinsic apoptosis activation pathways in an NF-κB-dependent manner. Immunoblot analysis of the expression levels of cleaved caspase-8 (18 and/or 43 kD), caspase-3 (17 kD), caspase-9 (37 kD), PARP (89 kD), as well as -tubulin (internal control) from total protein extracts (20 mg) of WT or stable mutant J774 cells mock-treated or infected by C. neoformans (KN99α) for the indicated time-points (n = 3). Arrowhead points out immunogenic specific band. (TIF) [file ppat.1002555.s007.tif]

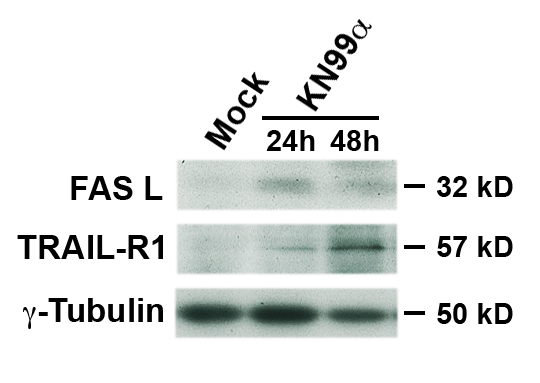

Supplement: Figure S8 — Fungal infection promotes FASL and TRAIL-R1/DR4 increase. Immunoblot analysis of the expression levels of FASL and TRAIL-R1/DR4, as well as γ-tubulin (internal control) from total protein extracts (30 mg) of J774 cells mock-treated or infected by C. neoformans (KN99α) for the indicated time-points (n = 2). (TIF) [file ppat.1002555.s008.tif]

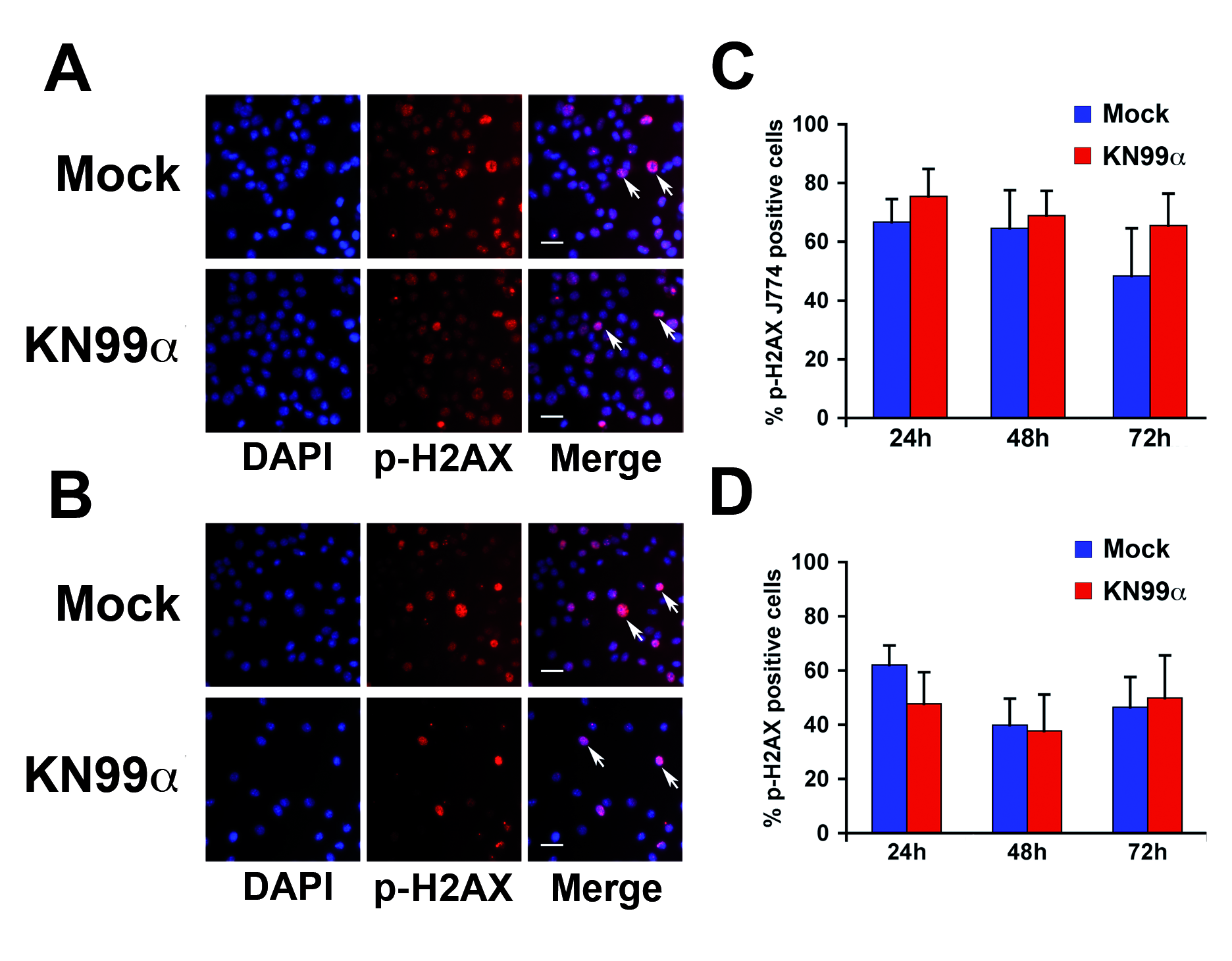

Supplement: Figure S9 — Absence of significant increase in γ-H2AX foci upon fungal infection. (A) Phospho-H2AX (p-H2AX or γ-H2AX) immunofluorescence and DAPI staining of mock-treated or C. neoformans-infected (KN99α) J774 cells 48 h p.i.. Scale bar 20 mm. (B) P-H2AX immunofluorescence and DAPI staining of mock-treated or C. neoformans-infected (KN99α) BMDM 48 h p.i.. Scale bar 20 µm. (C) Quantification of the number of p-H2AX foci per total number of cell nuclei (blue) in mock-treated or C. neoformans-infected (KN99α) J774 cells at the indicated time-points. Data are mean ± s.e.m. (nuclei; n = 300). (D) Quantification of the number of Phospho-H3+ nuclei per total number of cell nuclei (blue) in mock-treated or C. neoformans-infected (KN99α) BMDM at the indicated time-points. Data are mean ± s.e.m. (nuclei; n = 325). (TIF) [file ppat.1002555.s009.tif]
