# Supplementary material for: Fungal-Induced Cell Cycle Impairment, Chromosome Instability and Apoptosis via Differential Activation of NF-κB
Source: PLoS Pathog. 2012 Mar 1;8(3):e1002555. doi: 10.1371/journal.ppat.1002555 (PMC3291658; doi:10.1371/journal.ppat.1002555)
Supplement: Protocol S1 — Detailed Material and Methods used in this study. Cell culture and infection, Mice and infection, C. neoformans strains, Reichert differential interference contrast (DIC) imaging, Viability assay, Apoptosis assay, Immunohistochemistry, Cell cycle analysis by flow cytometry, Karyotyping cells, Electrophoretic mobility shift assay (EMSA), Reporter assay, Western blotting, Construction of J774 stable clones, Cell cycle sorting & Statistics. (DOC) [file ppat.1002555.s010.doc]

**Protocol S1**

**Cell culture and infection.** Murine macrophage J774.A1 cells (from the ATCC) were cultured in endotoxin-free Dulbecco’s modified Eagle’s medium with 10% decomplemented foetal bovine serum (PAA) at 37°C and 5% CO_2_. Bone Marrow Derived Macrophages (BMDM) were prepared from 7-9 weeks old female BALB/c or male *nfκb2*^–/–^ mice [1] and their C57BL/6 wild-type control, as reported [2] and cultured in endotoxin-free RPMI medium with 10% decomplemented fetal bovine serum (PAA), 50 μg/ml gentamicin and 30% CSF-conditioned medium from L929 cells at 37°C and 5% CO_2_. After 7 days of culture, adherent macrophages (BMDM) were recovered and directly used for experiments, i.e. without synchronization by CSF starvation. For infection, cells were plated at 2x10^6^ cells/10 cm diameter dish. 36 h later, medium was replaced by 10 ml of fresh medium with 2.5% serum; cells of a representative dish were counted and infection was performed at a M.O.I. of 5 yeasts/cell with the wild-type *C. neoformans* KN99α strain, unless otherwise specified. 2 ml of fresh medium was added after 48h. In case of MG132 administration, mock-treated or infected cells were treated 3h before recovery with 20 μM of MG132 (Sigma-Aldrich).

**Mice and infection.** Transgenic κB-*lacZ* mice [3] on a C57BL/6J × SJL mixed background have been described. Mice were housed in a specific-pathogen free animal house facility on a 12-h light/12-h dark cycle with ad libitum access to rodent chow. Animal handling and experimentation were performed in accordance with institutional guidelines and current French and EC legislation (law 87-848). Model of infection mimicking the systemic infection in humans has been described [4]. 2-4 months old mice were infected with a yeast inoculum of 1x10^5^ colony forming units (CFU) of wild-type *C. neoformans* strain, KN99α, in 200 μl of sterile PBS by intraveinous injection of tail vein. At indicated time, mice were euthanized; spleen was recovered using aseptic conditions, cut in 2 parts, one for immunohistochemistry treatment and one for determination of CFU; otherwise, alveolar macrophages were isolated as described [2], total extracts were prepared as previously reported [5] and purity of these cells was checked by FACS analysis with F4/80, GR1 and CD11b cell surface marker antibodies, whereas half-brain were used for CFU determination. For this former analysis, half-spleen or half-brain were weighed, and homogenized in 1 ml of sterile saline solution. Serial dilutions of homogenates were plated in duplicate onto Sabouraud agar petri dishes and CFU were enumerated after 48 h at 30°C.

***C. neoformans* strains.** Wild-type *C. neoformans* var. *grubii* strain (serotype A) KN99α and mutants (*cap59*D lacking GXM the major capsule component, two independent strains; *cas1*D lacking O-acetylation of the capsule; *uge1*D lacking GalXM and two suppressor strains of this capsule GalXM-deficient strain, *uge1*D supp1 and supp4, that have a doubling time close to that of WT strain at 37°C) have been described [6,7] as well as *C. neoformans* var. *neoformans* strain (serotype D) JEC21 [8]. To obtain reproducible NF-κB kinetics of activation, yeasts from 20% glycerol stocks stored at -80°C were spread onto Yeast Peptone Dextrose (YPD) plate and incubated for 48 h at 30°C. 10 ml of liquid YPD were inoculated with a full platinum handle of these fresh yeasts and incubated under microaerobic conditions on a rotary shaker for 19 h at 30°C. 10^7^ yeasts from this preculture were then used to inoculate 10 ml of YPD and incubated with agitation under microaerobic conditions at 30°C for 19 h. Yeasts were then harvested, washed three times in 50 ml phosphate-buffered saline pH 7.4 (PBS), resuspended in tissue culture medium with 2.5% foetal bovine serum for cell infection or in PBS for mice infection, and counted in a hematocytometer. For heat-inactivation experiment, yeasts in PBS were inactivated 15 min at 100°C, washed twice in 50 ml PBS and resuspended in tissue culture medium with 2.5% foetal bovine serum before addition to the cells.

**Reichert differential interference contrast (DIC) imaging.** J774 cells were seeded at 50000 cells/well on round coverglass in 24-well tissue culture plates. After infection, at indicated times, cells were rinsed twice in PBS, fixed in 4% paraformaldehyde in PBS for 10 min at 25°C, washed three times with PBS and video-enhanced (VE)-DIC microscopy was performed on a Reichert Polyvar2 (under a 40x/1.00 NA oil objective). VE-DIC images were generated using a 3-CCD video camera (HVD-25, Hitachi) and captured with the BTVPro software.

**Viability assay.** J774 cells or BMDM were seeded at 5000 and 20000 cells/well respectively in white opaque flat-bottomed 96-well tissue culture plates (Beckton Dickinson) in culture medium supplemented with 10% FCS. For transwell experiments, cells were seeded at 100000 cells/well in 12- well plates (Costar). 24h later cells were infected with *C. neoformans* in 2.5% FCS or placed in the upper chamber (3 mm membrane), in case of transwell experiments, and viability was assessed at indicated times according to the manufacturer’s recommendations by adding 70 μl (or 500 μl for transwell experiments) of CellTiter-Glo Luminescent cell viability assay reagent (Promega), generating a luminescent signal directly proportional to the amount of ATP present in metabolically active cells, and by measuring photon emission in 96-well Centro LB960 luminometer (Berthold). Data are representative of at least three independent experiments done in triplicate. Results are presented as fold relative to the activity of uninfected cells at t 0.

**Apoptosis assay.** Wild-type J774 cells, stable J774 clones or BMDM were seeded at 50000, 70000 and 100000 cells/well respectively on round coverglass in 24-well tissue culture plates. After infection, at indicated times, cells were rinsed twice in PBS, fixed in 4% paraformaldehyde in PBS for 10 min at 25°C, washed three times with PBS, permeabilized with 0.2% Triton, 0.1M sodium citrate 6 min on ice, washed twice in PBS and labelled by TUNEL assay (Roche). Images were acquired on an Axiovert 40 CFL microscope headcoupled to Axiovision v4.6.30 software (Zeiss) and interpretation of assays was done according to Galluzzi et al. [9].

**Immunohistochemistry.** Spleen tissue sections were processed for β-galactosidase activity detection as previously described [10]. In short, tissues were fixed in ethanol-aceton (70:30), dehydrated in xylene, and then embedded by three passages at 44°C in low melting point paraffin (Merck, VWR). For β-galactosidase activity detection, paraffin sections (7 μm) were incubated in 4-chloro-5-bromo-3-indoyl-b-galactoside (X-Gal) solution at 30°C for 16 h as reported [3], and either stained with Gomori-Grocott for yeast detection with safranin counterstaining or processed for immunohistochemistry. Primary monoclonal rat antibodies against mouse F4/80 (MF48000, Caltag, Invitrogen; 1:50) or MOMA-1 (3D6.1112 Abcam; 1:100) were used. Polyclonal rabbit anti-phospho-Histone H3 (Ser10) (9701, Cell Signaling Technology) antibody was applied at 1:100 dilution. Prediluted secondary antibodies (anti-rat IgG or anti-rabbit IgG coupled to streptavidin alkaline phosphatase or peroxydase conjugate; N-Histofine simple stain, Microm Microtech, Nichirei Biosciencecs Inc.) were used, giving rise after revelation to a blue staining for alkaline phosphatase and a red staining for peroxydase. Images were acquired on an Eclipse E800 microscope headcoupled to ACT-1 v.2 software (Nikon). Cultured cells were fixed 10 min in 4% paraformaldehyde, permeabilized for 15 min in 0.05% saponin, 0.1% NaN_3_, 1% BSA, and then incubated 16 h at 4°C with primary monoclonal mouse anti-human Ki-67 (550609, Beckton Dickinson; 1:10) or polyclonal rabbit anti-phospho-Histone H3 (Ser10) (9701, Cell Signaling Technology; 1:200) or monoclonal rabbit anti-human p-H2AX (9718, Cell Signaling Technology; 1:1000) antibodies. Anti-mouse IgG conjugated with Alexa Fluor 488 or anti-rabbit IgG conjugated with Alexa Fluor 555 (Invitrogen; 1:1000) were then added for 1h at 25°C. After DAPI counterstaining, immunoreactivities were visualized under an Axiovert 40 CFL microscope headcoupled to Axiovision v4.6.30 software (Zeiss).

**Cell cycle analysis by flow cytometry.** 5x10^6^-10^7^ cells in PBS were fixed by slow addition of 100% ethanol to a final concentration of 70% and stored at -20°C for at least 16 h. Fixed cells were then pelleted, washed twice in PBS and incubated for at least 30 min at 25°C with 200 mg/ml heat-inactivated pancreatic RNase A DNase free (Sigma). After centrifugation, cells were stained in 500 μl of 5 μg/ml propidium iodide and analysed by flow cytometry using a FACSCalibur and CellQuest software (Becton Dickinson). To make sure to get enough material for analysis, 30000 total events were acquired per sample at low rate. Identical parameters of acquisition were used for all samples, except when quantification of cells in the different phases of the cell cycle was meant. In that latter case, each sample was acquired with specific acquisition parameters, which arbitrarily positioned G0/G1 peak at 200 on the linear scale of FL2-A X-axis in order to allow cell cycle analysis by the software. For the analysis, gating was done by plotting the FL2-W (pulse-width) versus the FL2-A (pulse-area) in a dot plot as to exclude G1 doublets (which have a same FL2-A signal but increased FL2-W compared to single G2/M) as well as non specific low staining, corresponding to cell debris, aggregates and potential yeasts. In final, analysis was performed on gated cells corresponding to a single cell population. It should be stressed out that our experimental conditions, optimised for proper analysis of cell cycle, are not adapted to apoptotic cell detection (as leakage of damaged DNA from ethanol-fixed cells occurred during their hydration and subsequent staining); therefore no apoptotic peak was detected. Control reference cells, chicken red blood cells, were purchased from Charles River. Data presented are representative of at least three independent experiments, unless specified.

**Karyotyping cells.** Cells infected or not for 48h were treated with colchicine 0.1 μg/ml (Sigma) for 6h and treated as described in [11]. Briefly, after washes in PBS, cell pellet was resuspended gently in prewarmed 0.56% (w/v) KCl and incubated 15 min at 37°C. Cells were then fixed in methanol:glacial acetic acid (3:1) at 25°C and then spread on cleaned slides. For counting chromosomes, slide preparations were stained in Giemsa for 15 min and photographed with a 100x lens on an Axiovert 200 microscope (Zeiss) headcoupled to ACT-1 v.2 software (Nikon).

**Electrophoretic mobility shift assay (EMSA).** Nuclear or total extracts were prepared and bandshift assays were performed as previously reported [5] using the canonical NF-κB site derived from the promoter of the major histocompatibility complex class I H-2 K^b^ gene (KBF1) as a probe. To identify NF-κB complexes, a supershift assay was carried out by adding specific polyclonal sera raised against p65 (#1226), p50 (#1263), c-Rel (#1051), p52 (#1267) or RelB (C19, SantaCruz) to the reaction. Polyclonal sera raised against p50, p52, p65 and c-Rel were kind gifts of N. Rice (Frederick, MD).

**Reporter assay.** NF-κB-luciferase reporter construct [3] and EF1-*lacZ* [12] normalization vector were co-transfected into J774 cells using the V kit (Amaxa, Lonza). For induction, cells were infected for 4h or 24h with *C. neoformans* (KN99a). Cells were then lysed as described [12] and luciferase and b-galactosidase activities were measured using the luciferase and luminescent β-galactosidase kits (Promega and Clontech respectively) according to manufacturer’s recommendations with a 96-well Centro LB960 luminometer (Berthold). Luciferase activity was normalised to β-galactosidase activity and results, calculated as the mean ± s.e.m. from three independent experiments, are presented as fold relative to the activity of mock cells.

**Western blotting.** Total protein extracts were prepared as previously reported [5]. Protein samples were then separated by sodium dodecyl sulfate-polyacrylamide gel electrophoresis and transferred onto Immobilon-P (Millipore) as described previously [13]. The membranes were first blocked with 3% non-fat milk and then incubated with primary antibody in 0.3% non-fat milk. Immunoblots were then incubated with secondary antibodies coupled to horseradish peroxydase conjugate (goat anti-rabbit IgG, Biorad; 1:4000 or horse anti-mouse IgG; Vector Laboratories; 1:4000). Specific bands were revealed with the Pierce enhanced chemiluminescence system and exposed on X-Ray films (GE Healthcare). Primary antibodies against the following proteins were used: γ-tubulin (clone GTU-88, Sigma; 1:10000), NIK (#4994, Cell Signaling Technology; 1:500), Phospho-p100 Ser 866/870 (#4810, Cell Signaling Technology; 1:1000), p100 (#1495, N. Rice, Frederick; 1:1000) (cleaved caspase-3, -9 and PARP (apoptosis antibody sampler kit for mouse, Cell Signaling Technology; 1:1000), cleaved caspase-8 (9429, Cell Signaling Technology; 1:1000), cyclin-A (ab7956, Abcam; 1:250), cyclin-B1 (4138, Cell Signaling Technology; 1:1000), cyclin-D1 (clone SP4, Thermo Fisher Scientific; 1:500), cyclin-E (sc-481, Santa Cruz; 1:200), p27kip1 (610241, Becton Dickinson; 1:2500), Cdk2 (sc-6248, Santa Cruz; 1:200), cdk1(sc-54, Santa Cruz; 1:300), skp2 (sc-7164, Santa Cruz; 1:200), Mad2 (610679, Becton Dickinson; 1:1000), IκBα (sc-371, Santa Cruz; 1:500), anti-HA (HA-11, Eurogentec; 1:1000), anti-Flag M2 (F3165, Sigma; 1:3000), anti-FAS L (AB16982, Chemicon; 1:2500), anti-TRAIL R1/DR4 (06-744, Upstate Cell Signaling solutions; 1:1000).

**Construction of J774 stable clones.** Plasmids pRcCMV-superrepressor overexpressing IκBα S32AS36A [13], pRcCMV-HA-IKK1 DN overexpressing cDNA from IKK1 with S176A, S180A mutations [14], and plasmids IKK2 DA or DN with S177E, S181E or S177A, S181A mutations respectively [14] have been described. Not1 inserts containing IKK2 cDNA were subcloned in pcDNA3 containing a FLAG-tag and mutations were confirmed by sequencing. All plasmids were linearized in the vector sequence prior to transfection of J774 cells with the V kit (Amaxa, Lonza). Stable clones were selected and maintained at 400 mg/ml of geneticin. All experimental data shown here have been observed in at least two independent clones for each construct.

**Cell cycle sorting.** Cells infected or not for 48h, were extensively rinsed with PBS and then incubated with cell dissociation buffer (Invitrogen) for 20 min. Individualised cells (5x10^7^-10^8^) were rinsed in PBS, resuspended in Dulbecco’s modified Eagle’s medium with 10% fetal bovine serum and 20 mM Hepes PH7.4, counted and incubated at the concentration of 10^7^ cells/ml with 10 μg/ml of Hoechst 33342 (Sigma) for 25 min. Cells at the different phases of the cell cycle were immediately sorted on a Moflow cell sorter (Beckman Coulter) using the Summit software and kept on ice prior to total protein extract preparation.

**Statistics.** All values are expressed as means ± SEM. All experiments were performed at least three times in triplicate. Statistical analysis was done with the GraphPad Prism program, applying analysis of variance with ANOVA followed by Bonferroni’s post hoc test. Differences between values were considered significant at P ≤ 0.05.

**References**

1. Caamano JH, Rizzo CA, Durham SK, Barton DS, Raventossuarez C, et al. (1998) Nuclear factor (NF)-kappa-B2 (p100/p52) is required for normal splenic microarchitecture and B cell-mediated immune responses. J Exp Med 187: 185-196.

2. Zhang X, Goncalves R, Mosser DM (2008) The Isolation and characterization of murine macrophages. Curr Protocols in Immunol 83 14.11.11-14.11.14.

3. Schmidt-Ullrich R, Mémet S, Lilienbaum A, Feuillard J, Raphael M, et al. (1996) NF-κB activity in transgenic mice : developmental regulation and tissue specificity. Development 122: 2117-2128.

4. Charlier C, Chretien F, Baudrimont M, Mordelet E, Lortholary O, et al. (2005) Capsule Structure Changes Associated with Cryptococcus neoformans Crossing of the Blood-Brain Barrier. Am J Pathol 166: 421-432.

5. Feuillard J, Mémet S, Goudeau B, Lilienbaum A, Schmidt-Ullrich R, et al. (2000) In vivo identification of lymphocyte subsets exhibiting transcriptionally active NF-κB/rel complexes. Int Immunol 12: 613-321.

6. Moyrand F, Janbon G (2004) UGD1, encoding the Cryptococcus neoformans UDP-glucose dehydrogenase, is essential for growth at 37 degrees C and for capsule biosynthesis. Eukaryot Cell 3: 1601-1608.

7. Moyrand F, Fontaine T, Janbon G (2007) Systematic capsule gene disruption reveals the central role of galactose metabolism on Cryptococcus neoformans virulence. Mol Microbiol 64: 771-781.

8. Moore TD, Edman JC (1993) The alpha-mating type locus of Cryptococcus neoformans contains a peptide pheromone gene. Mol Cell Biol 13: 1962-1970.

9. Galluzzi L, Aaronson SA, Abrams J, Alnemri ES, Andrews DW, et al. (2009) Guidelines for the use and interpretation of assays for monitoring cell death in higher eukaryotes. Cell Death Differ 16: 1093-1107.

10. Ferrero RL, Ave P, Ndiaye D, Bambou JC, Huerre MR, et al. (2008) NF-kappaB activation during acute Helicobacter pylori infection in mice. Infect Immun 76: 551-561.

11. Nagy A, Gertsenstein M, Vintersten K, Behringer R (2003) Manipulating the mouse embryo. Cold Spring Harbor Laboratory Press Third Edition.

12. Kaltschmidt B, Ndiaye D, Korte M, Pothion S, Arbibe L, et al. (2006) NF-kappaB regulates spatial memory formation and synaptic plasticity through protein kinase A/CREB signaling. Mol Cell Biol 26: 2936-2946.

13. Fridmacher V, Kaltschmidt B, Goudeau B, Ndiaye D, Rossi FM, et al. (2003) Forebrain-specific neuronal inhibition of nuclear factor-kappaB activity leads to loss of neuroprotection. J Neurosci 23: 9403-9408.

14. Mercurio F, Zhu HY, Murray BW, Shevchenko A, Bennett BL, et al. (1997) IKK-1 and IKK-2 - cytokine-activated IκB kinases essential for NF-κB activation. Science 278: 860-866.
